# Structural Studies of Giant Empty and Endohedral Fullerenes

**DOI:** 10.3389/fchem.2020.607712

**Published:** 2020-12-03

**Authors:** Song Wang, Qing Chang, Guizhi Zhang, Fukun Li, Xingmin Wang, Shangfeng Yang, Sergey I. Troyanov

**Affiliations:** ^1^Chongqing Key Laboratory of Catalysis & Environmental New Materials, College of Environment and Resources, Chongqing Technology and Business University, Chongqing, China; ^2^Hefei National Laboratory for Physical Sciences at Microscale, Chinese Academy of Sciences (CAS) Key Laboratory of Materials for Energy Conversion, Department of Materials Science and Engineering, University of Science and Technology of China, Hefei, China; ^3^Department of Chemistry, Moscow State University, Moscow, Russia

**Keywords:** fullerene, giant fullerene, endohedral fullerene, chlorination, single crystal x-ray diffraction

## Abstract

Structure elucidations of giant fullerenes composed of 100 or more carbon atoms are severely hampered by their extremely low yield, poor solubility and huge numbers of possible cage isomers. High-temperature exohedral chlorination followed by X-ray single crystal diffraction studies of the chloro derivatives offers a practical solution for structure elucidations of giant fullerenes. Various isomers of giant fullerenes have been determined by this method, specially, non-classical giant fullerenes containing heptagons generated by the skeletal transformations of carbon cages. Alternatively, giant fullerenes can be also stabilized by encapsulating metal atoms or clusters through intramolecular electron transfer from the encapsulated species to the outer fullerene cage. In this review, we present a comprehensive overview on synthesis, separation and structural elucidation of giant fullerenes. The isomer structures, chlorination patterns of a series of giant fullerenes C_2*n*_ (2*n* = 100-108) and heptagon-containing non-classical fullerenes derived from giant fullerenes are summarized. On the other hand, giant endohedral fullerenes bearing different endohedral species are also discussed. At the end, we propose an outlook on the future development of giant fullerenes.

## Introduction

Giant fullerenes are those with 100 or more carbon atoms, namely, beginning with C_100_. They are always present in the pristine soot produced by arc-discharge or laser ablation of graphite, as well as C_60_, C_70_, and the higher fullerenes C_76_-C_98_ (Diederich and Whetten, 1991; Lamb et al., 1992). The prototype C_60_ is made up of 12 pentagons and 20 hexagons, possessing the perfect spherical structure (Kroto et al., 1985). Furthermore, empty fullerenes obey the isolated pentagon rule (IPR), namely, pentagons are surrounded by hexagons (Kroto, 1987). Giant fullerenes have been extracted from arc-generated carbon soot using solvents with different boiling points (Parker et al., 1991, 1992; Shinohara et al., 1992; Anacleto et al., 1993). Furthermore, the existence of giant fullerenes as large as C_500_ has been confirmed by mass spectrometry (MS) (Shinohara et al., 1992). The structures of the giant fullerenes become ever more complex as the number of carbon atoms increases. Various researchers have argued that the canonical form of the giant fullerenes is the bucky tube or sphere (Lamb et al., 1992). Subsequently, scanning tunneling microscope (STM) images have demonstrated that the giant fullerenes, extracted under high-pressure with toluene, are roughly spherical in shape and that their diameters fall in the range of ~1–2 nm, corresponding to fullerenes containing 60 to 330 atoms (Lamb et al., 1992). Theoretical calculations indicate that ball-shaped fullerenes are energetically favored over capsular (tube-like) fullerenes (Adams et al., 1992; York et al., 1994). In particular, the huge numbers of isomers and the extremely low yields of giant fullerenes make the identification of the structures of giant fullerenes extremely challenging. Since 2010, thanks to advanced separation technology and characterization methods, a series of giant fullerenes have been structurally resolved via exohedral chlorination. In addition, giant endohedral metallofullerenes (EMFs) have been investigated by co-crystallization with metalloporphyrins. Little by little, the uncertainties surrounding and the unknown characteristics of giant fullerenes are being resolved by detailed and exhaustive research efforts.

Exohedral chlorination of giant fullerenes provides an efficient way to identify the structures of these giant molecules. Usually, the pristine spherical fullerenes undergo a rotational/librational movement in crystals, so the structural elucidation of giant fullerenes is severely hampered. However, exohedral derivatization hinders the rotational/librational mobility of fullerene cages in the crystalline state. Exohedral chlorination involves chlorination, *in situ* crystal growth, and subsequent single-crystal diffraction to solve the structure of the giant fullerenes using synchrotron radiation (Troyanov and Kemnitz, 2012). Notably, this method is not only applicable to the individual isomers of fullerenes but sometimes also to fullerene mixtures. Dozens of giant fullerenes have been identified by chlorination, and the attachment patterns of chlorine atoms usually possess unique features contributing to stabilization of the chlorinated molecule. In particular, high-temperature chlorination of fullerenes can induce skeletal transformations that alter the carbon cage topology. Yang and Troyanov have discussed in detail the chlorination-promoted skeletal transformations of fullerenes in their recent review (Yang et al., 2019). As well, non-classical (*NC*) fullerenes containing seven-membered rings show unique structural characteristics compared to classic fullerenes containing only pentagons and hexagons (Qian et al., 2003; Tan et al., 2009). In this review, we focus on the isomer structures of giant fullerenes, and the attachment patterns of chlorine atoms. In particular, heptagon-containing non-classical giant fullerenes have also been described.

Endohedral fullerenes with atoms, ions, molecules, or clusters encapsulated in the fullerene cage exhibit specific structures and have great potential applications in quantum computing, biomedicine, and as magnetic materials (Popov et al., 2013; Cai et al., 2019; Feng et al., 2019; Chai et al., 2020). Giant EMFs were detected by MS, while their structural elucidation has been hindered. In 2009, Liu and Balch reported the isolation and structural characterization of the nanocapsule Sm_2_@*D*_3*d*_-C_104_(822) as the largest endohedral fullerene at that time, and its molecular structure was clearly identified by single-crystal X-ray diffraction (Mercado et al., 2009). Recently, Lu identified a series of giant metallic carbide fullerenes and extended the largest endohedral fullerene to Y_2_C_2_@C_106_ (Pan et al., 2018). In this review, we additionally summarize the giant endohedral metallofullerenes that have been reported and discuss their structural features.

## Synthesis, Separation, and Methodology

### Synthesis and Separation

Empty fullerenes are usually synthesized by a Krätschmer–Huffman DC-arc discharging method using a pure graphite rod under a helium atmosphere (Yang et al., 2012a). The carbon soot thus produced is extracted by solvents with different boiling points, including toluene, benzene, 1,2,3,5-tetramethylbenzene, 1,2,4-trichlorobenzene, pyridine (Parker et al., 1992), *N*-methyl-2-pyrrolidinone, quinoline, and carbon disulfide (CS_2_) (Parker et al., 1992; Shinohara et al., 1992; Anacleto et al., 1993). High-boiling solvents such as quinoline, are more efficient at extracting giant fullerenes. Shinohara reported that giant fullerenes were extracted by quinoline, with molecular formulas up to C_500_ were confirmed by MS (Shinohara et al., 1992). Furthermore, Soxhlet extraction of fullerenes performed much better than simple reflux, and resulted in extraction yields that were almost twice as high, which was confirmed by Parker et al. (1992). Moreover, Müllen et al. reported that using a reactive extraction for the as-produced soot with 5-hexadecanamido-1,3-dihydro-2-benzothiophene 2,2-dioxide, an *ortho*-quinodimethane precursor, soluble materials consisting of multiple adducts of fullerenes C_60_-C_418_ were achieved (Beer et al., 1997).

In contrast, endohedral fullerenes are synthesized by an improved Krätschmer–Huffman DC-arc discharging method with a doped drilled graphite rod that is filled with mixtures of rare-earth oxides and graphite powders (Liu et al., 2016). Giant fullerenes were extracted from the as-produced fullerene soot by ultrasonic extraction with *o-*dichlorobenzene (ODCB) or Soxhlet extraction with 1,2,4-trichlorobenzene (TCB) under a nitrogen atmosphere (Mercado et al., 2009; Pan et al., 2018). Solvents with high boiling points such as TCB are preferred due to the lower solubility of giant endohedral fullerenes compared to giant empty fullerenes.

The separations of giant fullerenes rely heavily on high-performance liquid chromatography (HPLC) with a series of specialized chromatographic columns. In addition, recycling HPLC is a prerequisite because of the similar retention times for adjacent giant fullerenes and the isomers of giant fullerenes. Taking the separation of C_100_ as an example, the extracted fullerene mixture was first subjected to HPLC separation using a preparative 5PYE column with toluene as the mobile phase (Yang et al., 2014a). Then, the subfraction eluting between 41.4 and 44.6 min was isolated by a semi-preparative Buckyprep column, and the main subfractions were then subjected to recycling HPLC separated with a semi-preparative Buckyprep-M column (Yang et al., 2014a). On the basis of MS analyses, three subfractions containing a prevalence of C_100_ were collected after several separation cycles (Yang et al., 2014a). Finally, the purest C_100_ subfraction was used as the starting material for chlorination. In consequence, the isolation of C_100_ involves three steps with specialized chromatographic columns and several recycles. This makes it time-consuming to acquire pure isomers of giant fullerenes, which seriously hinders their structural elucidation.

Very recently, Koenig et al. reported a creative method to isolate fullerenes with tubular shape (fullertubes) by two stages (Koenig et al., 2020). In brief, 500 mg of arc-generated soot extract was dissolved in 500 mL of toluene (1 mg/mL), then 15 mL of 3-amino-1-aminopropanol was added with stirring; after stirring for an hour, reaction mixture became two layers: the organic phase containing unreacted fullertubes and the aqueous layer (bottom) having reacted spheroidal fullerene contaminants; by thoroughly washing and rotary evaporation, 38 mg of sample enriched in fullertubes was obtained (Koenig et al., 2020). Then, at the second stage, the sample was isolated by one stage of HPLC only, and several purified fullerenes with tubular shape were acquired including *D*_5*h*_-C_90_(1), *D*_3*d*_ -C_96_(3) and *D*_5*d*_-C_100_(1) (Koenig et al., 2020). In especial, when the toluene was replaced by xylenes with better solubility for fullerenes, 42 mg sample was achieved. By further HPLC separation, purified samples of C_108_, C_120_, C_132_, and C_156_ were obtained for the first time (Koenig et al., 2020). This method combining high-efficiency chemical separation and HPLC purification represents a new approach to enrich and isolate the giant fullerenes.

### Chlorination and Crystal Growth

In the chlorination experiment, the subfraction containing the empty giant fullerene was placed in a glass ampoule together with chloride reagents such as VCl_4_, SbCl_5_ or their mixtures. The ampoule was then evacuated, sealed off, and heated at 350–360°C for several days or weeks until crystals of chlorinated derivative had formed. After washing out the excess chloride reagents with HCl and water, small crystals remained behind whose crystallographic properties were acquired *in situ* by synchrotron radiation single-crystal X-ray diffraction and which unambiguously revealed the molecular structure of the giant fullerenes.

However, this standardized exohedral method is unsuitable for endohedral fullerenes. Notably, chlorination of the latter does not proceed, although many attempts have been made. The alternative method is co-crystallization using Ni(OEP) (OEP = 2,3,7,8,12,13,17,18-octaethylporphin dianion) as the host (Stevenson et al., 1999). The co-crystallization of giant endohedral metallofullerenes with Ni(OEP) usually provides suitable crystals for X-ray diffraction and is achieved by slow diffusion of a toluene solution of a giant endohedral metallofullerene into a toluene solution of Ni(OEP). Volatile solvents, including benzene and CS_2_, can be used as alternatives to dissolve the giant fullerene (Wei et al., 2016). Surprisingly, in the case of La_2_C_2_@*D*_5_-C_100_(450), La_2_C_2_@*Cs*-C_102_(574), and La_2_C_2_@*C*_2_-C_104_(816), only the fullerene and the intercalated CS_2_ molecules are present, while the Ni(OEP) and other solvents are absent (Cai et al., 2015, 2016).

### Theoretical Calculations

Theoretical calculations also play an important role in probing the structures of pristine giant fullerenes (Yoshida et al., 1996; Achiba et al., 1998; Zhao et al., 2004; Cai et al., 2005; Shao et al., 2006, 2007). Giant empty fullerenes obey the IPR, while the number of isomers of giant fullerenes beyond C_100_ is enormous. It is impossible to accurately optimize entire isomers of the giant fullerenes; therefore, viable strategies have been put forward for achieving this. Usually, prescreening tools such as the IPR, the hexagon-neighbor rule (HNR), or the approximate standard enthalpy formula first reduce the number of candidate isomers (Cai et al., 2005). Then, an efficient screening tool such as the empirical force field method or semi-empirical methods further reduces the number of low-energy candidates (Cai et al., 2005). Based on these methods, giant fullerenes up to C_120_ have been studied and the lowest-energy structures have been predicted (Yoshida et al., 1996; Achiba et al., 1998; Zhao et al., 2004; Cai et al., 2005; Shao et al., 2006). Almost identical optimized structures for the same giant fullerene were achieved by ab initio quantum chemistry or density functional theory (DFT) calculations; however, there are still some deviations from the predictions (Zhao et al., 2004; Cai et al., 2005; Shao et al., 2006).

For chlorinated derivatives of giant fullerenes, theoretical calculations of their formation energies on the DFT level reveal that the average enthalpy of chlorine addition (calculated per Cl atom) decreases monotonically with increasing number of attached Cl atoms, which is similar to the behavior of the reported chloro-derivatives of higher fullerenes (Papina et al., 2007; Troyanov and Kemnitz, 2012). More favorable aromatic substructures have been formed by chlorine addition, notably contributing to the stabilization of the chlorinated derivative (Troyanov and Kemnitz, 2012; Yang et al., 2019). Furthermore, the Stone–Wales rotation (SWR) and C2L mechanisms of skeletal transformations of fullerenes are presented in Yang and Troyanov's recent detailed review (Yang et al., 2019).

So far, few theoretical calculations of giant endohedral metallofullerenes have been conducted. According to the sizeable (LUMO-4)–(LUMO-3) gap and the formal transfer of six electrons to the cages, Poblet has proposed the most stable structures for the six higher endohedral metallofullerenes from C_92_ to C_100_ (Valencia et al., 2007). However, no exact theoretical calculations for other models with different charge transfer properties and other giant fullerenes beyond C_100_ have been reported.

## Structural Studies of Giant Fullerenes

### Isomer Structures and Chlorination Patterns

So far, isomer structures and chlorination patterns of giant fullerenes from C_100_ to C_108_ have been summarized in Table 1 and detailed discussions are presented in the following page.

**Table 1:** Isomer structures and chlorination patterns of giant fullerenes from C_100_ to C_108_.

**Giant fullerenes**	**Isomer**	**Chlorinated derivative**	**Stabilized substructure**	**References**
C_100_	*C_2_*-C_100_(18)	C2-C_100_(18)Cl_28/30_	Four (nearly)isolated C=C double bonds and two ethenylbenzene substructure	(Wang et al., 2016a)
	*D_5*d*_*-C_100_(1)	*C_2*h*_*-C_100_(1)Cl_12_	Two butadiene-like fragments	(Fritz et al., 2014)
	C1-C_100_(425)	*C_1_*-C_100_(425)Cl_22_	Three isolated C=C double bonds and two benzenoid ring	(Wang et al., 2016a)
	*C_2*v*_*-C_100_(417)	*C_*s*_*-C_100_(417)Cl_28_	Two butadiene-like substructures and two aromatic systems within coronene units	(Wang et al., 2016a)
C_102_	C_102_(19)	^#283794^C_102_Cl_20_	A biphenyl-like substructure	(Yang et al., 2013)
	C_102_(603)	C_102_(603)Cl_18_	Two benzenoid rings and two isolated C=C double bonds	(Yang et al., 2014b)
		C_102_(603)Cl_20_	Three (nearly isolated) benzenoid ring and two nearly isolated C=C double bonds	(Yang et al., 2014b)
C_104_	C_104_(258)	*C_1_*-C_104_(258)Cl_16_	A nearly benzenoid-like fragment and an isolated C=C double bond	(Yang et al., 2014c)
	C_104_(234)	C_104_(234)Cl_16−22_	The number of isolated C=C double bond correspond to 3,4,5, and 6, respectively	(Yang et al., 2014b)
	*D_2_*-C_104_(812)	*D_2_*-C_104_(812)Cl_24_	Four isolated benzenoid rings and four C=C double bonds	(Yang et al., 2014c; Jin et al., 2017)
		*D_2_*-C_104_(812)Cl_12_	None	(Jin et al., 2017)
	C_104_(811)	*C_2_*-C_104_(811)Cl_24_	Four isolated C=C double bonds and four benzenoid rings	(Yang et al., 2014c)
		*C_2_*-C_104_(811)Cl_28_	Two nearly isolated benzenoid rings and four isolated C=C double bonds	(Jin et al., 2017)
C_106_	C_106_(1155)	C_106_(1155)Cl_24_	Four isolated C=C double bonds and seven entirely or nearly isolated, benzenoid rings	(Wang et al., 2016b)
C_108_	*D_2_*-C_108_(1771)	*C_2_*-C_108_(1771)Cl_12_	None	(Wang et al., 2016b)

#### C_100_

So far, several isomers of C_100_ have been identified via chlorination followed by studies of single-crystal X-ray diffraction, including *C*_2_-C_100_(18), *D*_5*d*_-C_100_(1), *C*_1_-C_100_(425), and *C*_2*v*_-C_100_(417) [isomer numbering according to the spiral algorithm (Fowler and Manolopoulos, 1995)]. *C*_2_-C_100_(18) is the first isomer of C_100_ disclosed by structure reconstruction, although theoretical calculations for all 450 IPR isomers of C_100_ indicate that C_100_(18) ranks second and follows the most stable *D*_2_-C_100_(449) (Zhao et al., 2004). However, *C*_2_-C_100_(18) was even excluded from the list of rather stable isomers in other theoretical calculations, whereas *D*_2_-C_100_(449) is still the most stable isomer (Cai et al., 2005; Shao et al., 2006). Interestingly, on the basis of theoretical calculations*, D*_5*d*_-C_100_(1), having a much higher relative formation energy, should therefore be highly unstable, but it has been captured by chlorination (Fritz et al., 2014).

**Figure 1:**
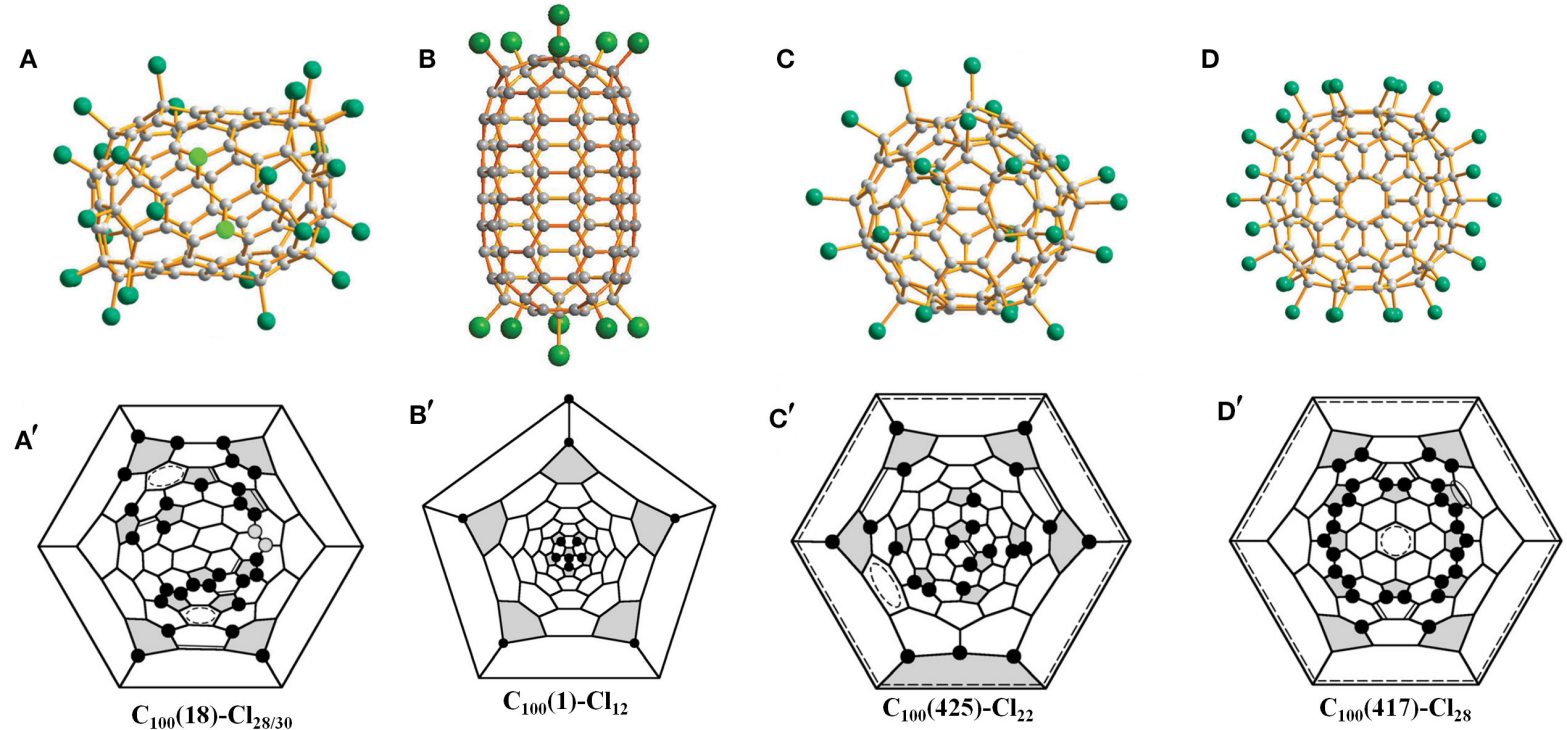
Projections and Schlegel diagrams of: **(A,A′)** C_100_(18)C_l28/30_; **(B,B′)** C_100_(1)Cl_12_; **(C,C′)** C_100_(425)Cl_22_; and **(D,D′)** C_100_(417)Cl_28_.

Chlorination of C_100_ fullerene afforded a non-classical fullerene chloride, C_96_Cl_20_, containing three heptagons (Yang et al., 2014a). Using structural reconstruction, *C*_2_-C_100_(18), out of 450 topologically possible IPR isomers, was established as the starting fullerene (Yang et al., 2014a). Using the same C_100_(18) fullerene as the starting material, another non-classical fullerene chloride, C_94_(*NC*1)Cl_22_, containing one heptagon together with the aforementioned C_96_(*NC*3)Cl_20_ were unexpectedly obtained (Ioffe et al., 2015). The detailed structural features and transformation mechanisms are presented in Section Heptagon-containing fullerenes derived from giant fullerenes below. Fortunately, the pristine C_100_(18) was directly captured by chlorination as C_100_(18)Cl_28/30_ (Figure 1A) without any cage shrinking; hence, the C_100_(18) was reconfirmed to exist in the as-produced fullerene soot (Wang et al., 2016a). Notably, C_100_(18)Cl_28/30_ is produced in a relatively short reaction time of about a week, while the cage transformation needs a longer reaction time (Wang et al., 2016a). This indicates that the reaction time for chlorination plays a vital role in determining the ultimate chlorination products. As shown in the Schlegel diagrams (Figure 1A′), relatively long chains of adjacent (*ortho*) attachments of Cl atoms are formed in regions of two closely arranged groups of four pentagons (Wang et al., 2016a). However, due to the two additional Cl atoms attached at triple hexagon junctions (THJs), which are usually unfavorable positions for fullerenes, a longer *ortho* chain of Cl atoms appears in C_100_(18)Cl_30_ (Wang et al., 2016a). Two ethenylbenzene-like substructures and four isolated and nearly isolated C=C double bonds boost the stability of the structure (Wang et al., 2016a). Notably, the chlorination patterns of *C*_2_-C_100_(18)C_l28/30_ are remarkably different from the assumed chlorination patterns for *C*_2_-C_100_(18)Cl_24_, which is regarded as the pristine structure of the cage transformations to C_96_(*NC*3)Cl_20_ and C_94_(*NC*1)Cl_22_. The possible reason for this is that, in further reactions, the chlorination pattern may change to structures inclining toward skeletal transformations via a “chlorine dance.” (Wang et al., 2016a).

*C*_2*h*_-C_100_(1)Cl_12_ (Figure 1B), which contains an unprecedented nanotubular carbon cage with the symmetry of highly unstable *D*_5*d*_, has been reported by Troyanov (Fritz et al., 2014). The crystal of C_100_(1)Cl_12_ was distinguished from a complex mixture of chlorinated fullerenes, and similar cases were observed for the crystallization of more than one chloride from a fullerene mixture possessing different molecular shapes (Fritz et al., 2014). *C*_2*h*_-C_100_(1)Cl_12_ displays a remarkable tube-like molecular shape because of a unique distribution of 12 pentagons on two poles of the *D*_5*d*_-C_100_(1) cage (Fritz et al., 2014). In detail, in each group formed by six pentagons on its poles, a central pentagon on the *C*_5_ axis is surrounded by five other pentagons, which is similar to the cases of the C_60_ and C_70_ molecules. Therefore, according to the similarity to C_60_, the chlorination pattern of *D*_5*d*_-C_100_(1)Cl_12_ (Figure 1B′), on both poles, adopts the skew-pentagonal pyramidal (SPP) arrangement, which is identical to the addition pattern of the *C*_*s*_-C_60_Cl_6_ and *C*_*s*_-C_60_(CF_3_)_12_ (Shustova et al., 2006; Omelyanyuk et al., 2007). Apparently, C_100_(1) possessing fragments of the C_60_ cage on each pole reacts easily under conditions of higher temperatures to form C_100_(1)Cl_12_; however, further chlorination may become slower because no unoccupied pentagons exist and, very likely, the chlorination product precipitates because of crystallization (Fritz et al., 2014). In fact, *D*_5*d*_-C_100_(1) was not expected to be present in fullerene soot on the basis of its much higher relative formation energy; that is, it should be highly unstable (Zhao et al., 2004). A plausible reason for *D*_5*d*_-C_100_(1) remaining in the carbon soot is that the distinctive features of the *D*_5*d*_-C_100_(1) cage prevent it transforming into more stable IPR isomers of C_100_ during fullerene synthesis (Fritz et al., 2014).

Another chlorination experiment on the subfraction containing C_100_ affords two crystalline modifications of C_100_(425)Cl_22_, while their crystal structures are different from only the packing motifs (Wang et al., 2016a). The C_100_(425)Cl_22_ molecule presents a rather spherical shape (Figure 1C) compared with C_100_(18)Cl_28/30_ because of the absence of coronene substructures in the cage (Wang et al., 2016a). As shown in Figure 1C′, the chlorination patterns of C_100_(425)Cl_22_ contain two sets of Cl attachments in adjacent positions (Wang et al., 2016a). As a result, three isolated C=C double bonds and two benzenoid rings contribute to the stabilization of the chlorination patterns (Wang et al., 2016a).

In addition, isomer *C*_2*v*_-C_100_(417) was confirmed via the chlorinated derivative *C*_*s*_-C_100_(417)Cl_28_ in two crystal structures (Wang et al., 2016a). One structure is made up of symmetrical mirror molecules and *C*_*s*_-C_100_(417)Cl_28_ appears observably flattened because there are two coronene substructures on opposite sides of the carbon cage (Figure 1D) (Wang et al., 2016a). Simultaneously, 26 attached Cl atoms are settled on the basis of the *C*_2*v*_ symmetry of the cage, whereas the symmetry of the entire chlorinated molecule is reduced to *C*_*s*_ because of the two attached Cl atoms, as shown in Figure 1D′ (Wang et al., 2016a). Furthermore, two butadiene-like substructures and two aromatic systems within coronene units have formed in the carbon cage of C_100_(417)Cl_22_ (Wang et al., 2016a). In the other crystal structure, C_100_(417)Cl_28_ and C_98_(*NC*1)Cl_26_ have co-crystallized in the same crystallographic site with 0.471 and 0.529 occupancies, respectively. A comparison of the Schlegel diagrams for the two molecules proved that a heptagon in the carbon cage of the chloride C_98_ stemmed from the loss of a 5:6 C–C bond of C_100_(417)Cl_28_ (Wang et al., 2016a).

#### C_102_

So far, two isomers, C_102_(19) and C_102_(603), among the 616 topologically possible IPR isomers of C_102_ have been identified by exohedral chlorination. On the basis of theoretical calculations, C_102_(603) was predicted as the most stable isomer, whereas C_102_(19) has lower stability due to its relatively lower formation energy compared with those of the other giant fullerenes.

**Figure 2:**
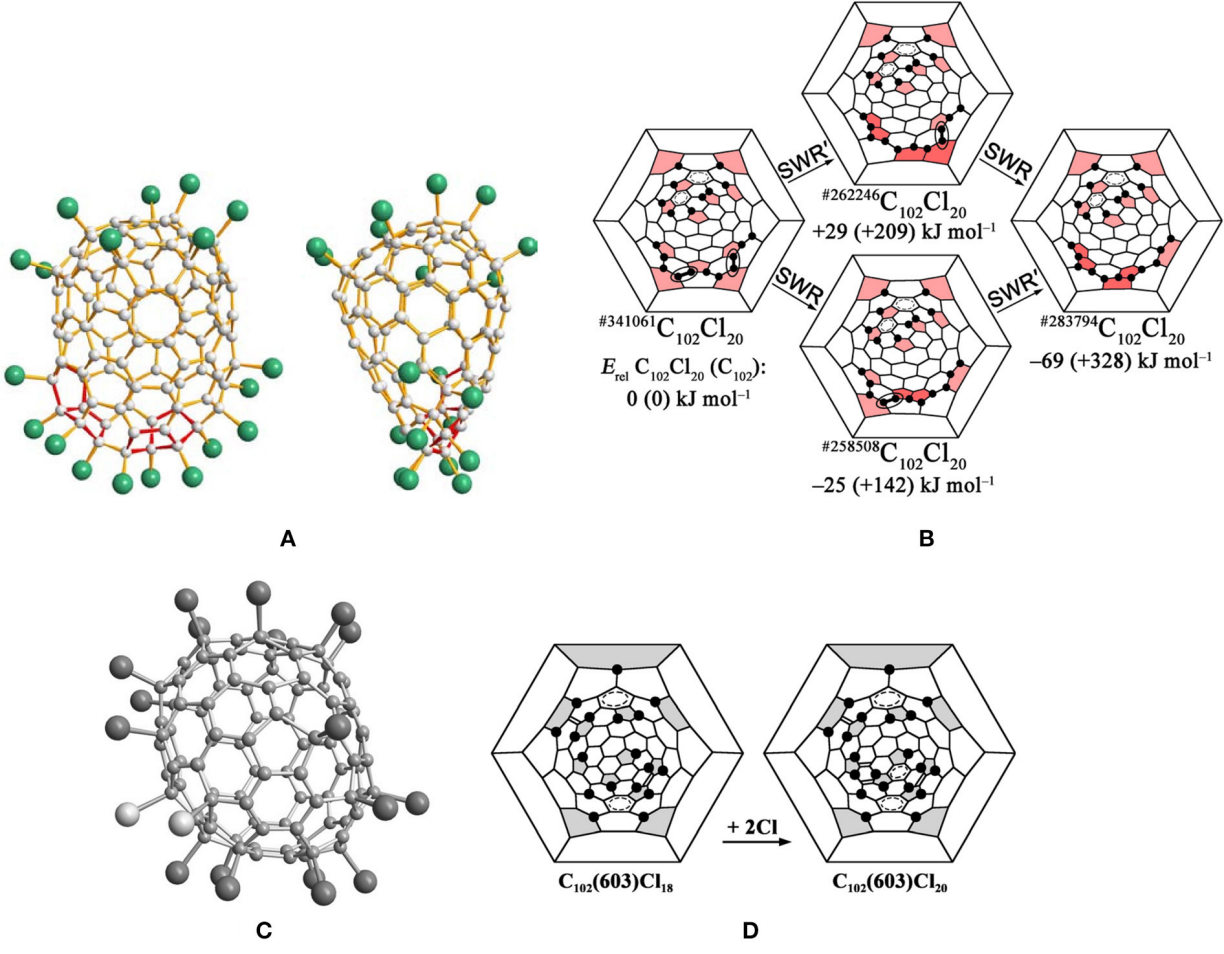
**(A)** Two mutually perpendicular projections of the ^#283794^C_102_Cl_20_ molecule; **(B)** Schlegel diagram description of the possible pathways of SWR transformations of the IPR ^#341061^C_102_Cl_20_ into the non-IPR ^#283794^C_102_Cl_20_; **(C)** perspective view of the C_1_-C_102_(603)Cl_18_/20 molecules. Two additional Cl atoms for C_102_(603)Cl_20_ highlighted in light gray; **(D)** Schlegel diagrams of C_102_(603)Cl_18_ and C_102_(603)Cl_20_.

The first identified isomer of C_102_ was C_102_(19) in 2013, which has been confirmed by the structural reconstruction of the obtained non-IPR fullerene chloride C_102_Cl_20_ (Yang et al., 2013). There are two pairs of fused pentagons on the sharpened cage end of the cage of C_102_Cl_20_, while the other end of the cage looks rather roundish (Yang et al., 2013), as shown in Figure 2A. However, two opposite sides of the carbon cage are significantly flattened (Yang et al., 2013). The non-IPR C_102_ isomer is assigned as No. 283794 [according to the spiral algorithm (Fowler and Manolopoulos, 1995)] among 341,658 topologically possible classical isomers of C_102_, which contains five- and six-membered rings only (Yang et al., 2013). In ^#283794^C_102_Cl_20_, five pentagons with two neighboring pairs of fused pentagons are closely located, in contrast to the seven residual pentagons situated far from them (Figure 2B) (Yang et al., 2013). In the cage area of the former, 11 Cl atoms occupy adjacent positions to the carbon atoms and form a long zigzag chain on the carbon cage (Yang et al., 2013). In the area of the seven dispersed pentagons, nine Cl atoms are primarily attached at *para* positions of the cage hexagons (and one *ortho* position), resulting in a biphenyl-like substructure formed by two pseudo-aromatic rings (Yang et al., 2013). However, the carbon cage shows a flattened shape due to two groups of fused hexagons (coronene substructures) existing in the regions between the two groups of chlorine attachments (Yang et al., 2013).

The reason for the formation of the non-IPR ^#283794^C_102_Cl_20_ is Stone–Wales (SW) transformations promoted by chlorination on the basis of the assumptions that the actual pathway has a minimum number of rearrangement steps and the IPR–IPR transformations do not occur at the reaction temperature (Yang et al., 2013). The IPR C_102_ fullerene No. 19, corresponding to No. 341,061 in the list of all classical C_102_ cages, has been confirmed as the starting isomer by structural reconstruction, and suffered only two SW rearrangement steps to obtain the non-IPR chloride (Yang et al., 2013). As shown in Figure 2B, the skeletal transformation of chlorinated ^#341061^C_102_ to ^#283794^C_102_Cl_20_ can be formally realized via two alternative chlorinated intermediates, ^#262246^C_102_Cl_20_ or ^#258508^C_102_Cl_20_, dependent on the order of SWRs of the two chlorinated C–C bonds in ^#341061^C_102_Cl_20_ (Yang et al., 2013). The DFT calculations demonstrate that ^#258508^C_102_Cl_20_ is the more possible intermediate on the path from the IPR ^#341061^C_102_ to ^#283794^C_102_Cl_20_ (the SWR–SWR′ pathway), which is comparatively more stable than ^#262246^C_102_Cl_20_. Fortunately, in 2018, the intermediate ^#258508^C_102_Cl_20_ was captured in co-crystals with the ultimate ^#283794^C_102_Cl_20_ (Mazaleva et al., 2018). Moreover, the relative energies of the two paths have been updated, which also sustains the SWR–SWR′ pathway, as shown in Figure 2B (Mazaleva et al., 2018).

The most stable IPR isomer, C_102_(603), on the basis of DFT calculations, was captured by its chloride, C_102_(603)Cl_18/20_, in 2014, as shown in Figure 2C (Yang et al., 2014c). Furthermore, the C_102_(603)Cl_18_ and C_102_(603)Cl_20_ molecules co-crystallize in the same crystallographic site with an occupancy ratio of 63/37 (Yang et al., 2014c). As shown in Figure 2D, the attachment of Cl atoms of C_102_(603)Cl_18_ featured in *para* positions in cage hexagons leads to the formation of two stabilizing benzenoid rings and two isolated C=C double bonds (Yang et al., 2014c). Unusually, a carbon atom in the position of a THJ leading to more planar arrangements of C–C bonds, which is generally unfavorable for addition in fullerenes, is attached by one Cl atom. In the case of C_102_(603)Cl_18_, such an uncommon attachment site is most likely induced by the formation of an isolated quasi-aromatic substructure on the cage (Yang et al., 2014b). Furthermore, achieving C_102_(603)Cl_20_ by two additional Cl atoms attached to C_102_(603)Cl_18_ is favored because of the formation of the third (nearly isolated) benzenoid ring and two nearly isolated C=C double bonds (Yang et al., 2014b).

#### C_104_

Four isomers of the giant fullerene C_104_, named as C_104_(258), C_104_(812), C_104_(234), and C_104_(811) [according to the spiral algorithm (Fowler and Manolopoulos, 1995)], have been successively confirmed by chlorination (Yang et al., 2014b,c; Jin et al., 2017). They have different stabilities according to the DFT calculations, namely, the most stable isomer, C_104_(234), a rather unstable isomer, C_104_(258), a moderately stable isomer C_104_(812), and much less stable isomer, C_104_(811).

**Figure 3:**
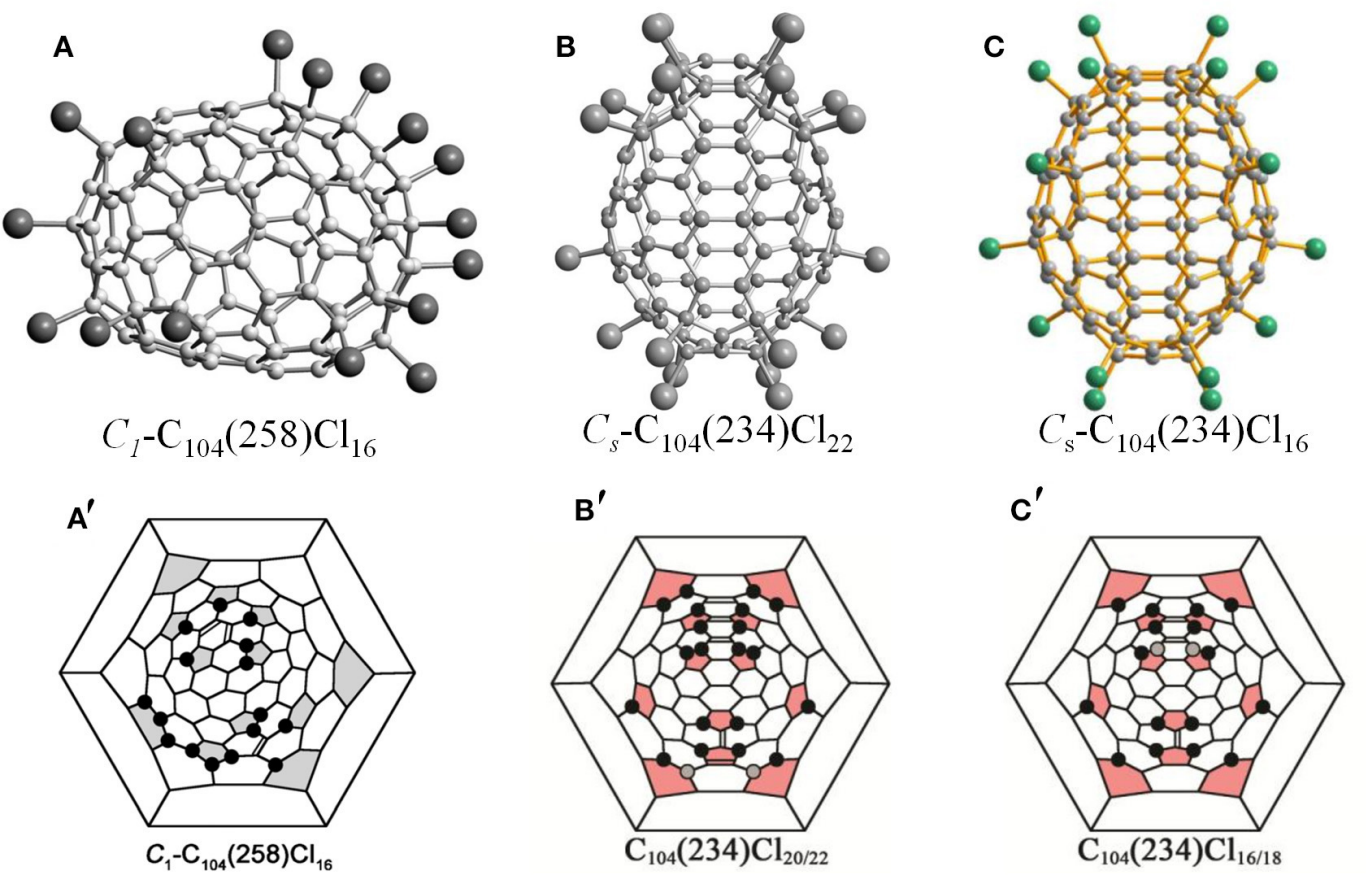
Projections and Schlegel diagram of: **(A,A′)** *C*_1_-C_104_(258)Cl_16_; **(B)** projections of *C*_*s*_-C_104_(234)Cl_22_; **(B′)** Schlegel diagram of *C*_*s*_-C_104_(234)Cl_20/22_; **(C)** projections of *C*_*s*_-C_104_(234)Cl_16_; and **(C′)** Schlegel diagram of *C*_*s*_-C_104_(234)Cl_16_.

Isomer *C*_1_-C_104_(258) has first been captured as the chloride, C_104_Cl_16_, which displays an elongated barrel-like shape because of the distribution of pentagons on opposite sides of the cage (Figure 3A) (Yang et al., 2014c). Due to several areas of annulated hexagons (like coronene substructures) existing between the groups of pentagons, the cage appears flattened (Yang et al., 2014c). As shown in Figure 3A′, the molecule structure of C_104_Cl_16_ is distinguished by the Cl atom attachments on the opposite ends of the cage, which contains pentagons, whereas the middle parts remain without any Cl atoms attached (Yang et al., 2014c). A nearly benzenoid-like fragment on the cage is isolated by six Cl atoms, whereas an isolated C=C double bond is formed by Cl atoms in four *para*-positions, and a six-membered chain of adjacent Cl atom additions occurs in the area adjacent to the isolated C=C double bond (Yang et al., 2014c). In particular, the feature of the chlorination pattern in *C*_1_-C_104_(258)Cl_16_ is that 16 Cl atoms (i.e., more than the 12 addends) occupy 10 pentagons, whereas two remaining pentagons are spare (Yang et al., 2014c). This is similar to the case of C_88_(17)Cl_16_, which presents a non-uniform attachment of Cl atoms, with two spare cage pentagons remaining (Yang et al., 2012b).

The most stable isomer of the giant fullerene C_104_, C_104_(234), has been confirmed by chlorination followed by a single-crystal diffraction study, and two crystal structures of C_104_(234)Cl_17.3_ and C_104_(234)Cl_22_ provide information regarding the chlorination patterns of C_104_(234) in the range of C_104_(234)Cl_16−22_, as shown in Figures 3B,C (Yang et al., 2014b). Three overlapping molecules of C_104_(234)Cl_16_, C_104_(234)Cl_18_, and C_104_(234)Cl_20_ co-crystallize in the same crystallographic site, while *C*_*s*_-C_104_(234)Cl_22_ solely forms another crystal (Yang et al., 2014b). C_104_(234)Cl_16−22_ molecules are mirror symmetrical, which corresponds to the pristine *C*_*s*_-C_104_(234) cage (Yang et al., 2014b). In the *C*_*s*_-C_104_(234)Cl_16_ molecule (Figure 3C′), each pentagon initially has one Cl atom, then four Cl atoms additionally attach at the 1,3-position of the four pentagons, which leads to the formation of two isolated C=C double bonds (Yang et al., 2014b). Simultaneously, the chlorination pattern is made up of four similar *para*-chains. However, further *para*-chain propagation is forbidden, because the only option is the unfavorable THJ site for *para*-chain propagation (Yang et al., 2014b). Therefore, *ortho* positions, despite the existing steric strain, are further occupied by pairs of Cl atoms, which is energetically more favorable because an extra isolated C=C double bond is formed. In the end, as shown in Figures 3B′,C′, three, four, and five C=C double bonds form in the carbon cages with 18, 20, and 22 attached Cl atoms, respectively (Yang et al., 2014b; Jin et al., 2017). Later, another chloro-derivative, *C*_*s*_-C_104_(234)Cl_16.78_, was reported, and its structure is close to the structure of *C*_*s*_-C_104_(234)Cl_17.26_ (Jin et al., 2017). The differences between the structures of *C*_*s*_-C_104_(234)Cl_16.78_ and *C*_*s*_-C_104_(234)Cl_17.26_ originate only from occupancy ratios of the molecules with 16, 18, and 20 attached Cl atoms, namely, 65/31/4 and 47/43/10, respectively (Jin et al., 2017). However, their crystallographic symmetries and packing motifs are also different (Jin et al., 2017).

**Figure 4:**
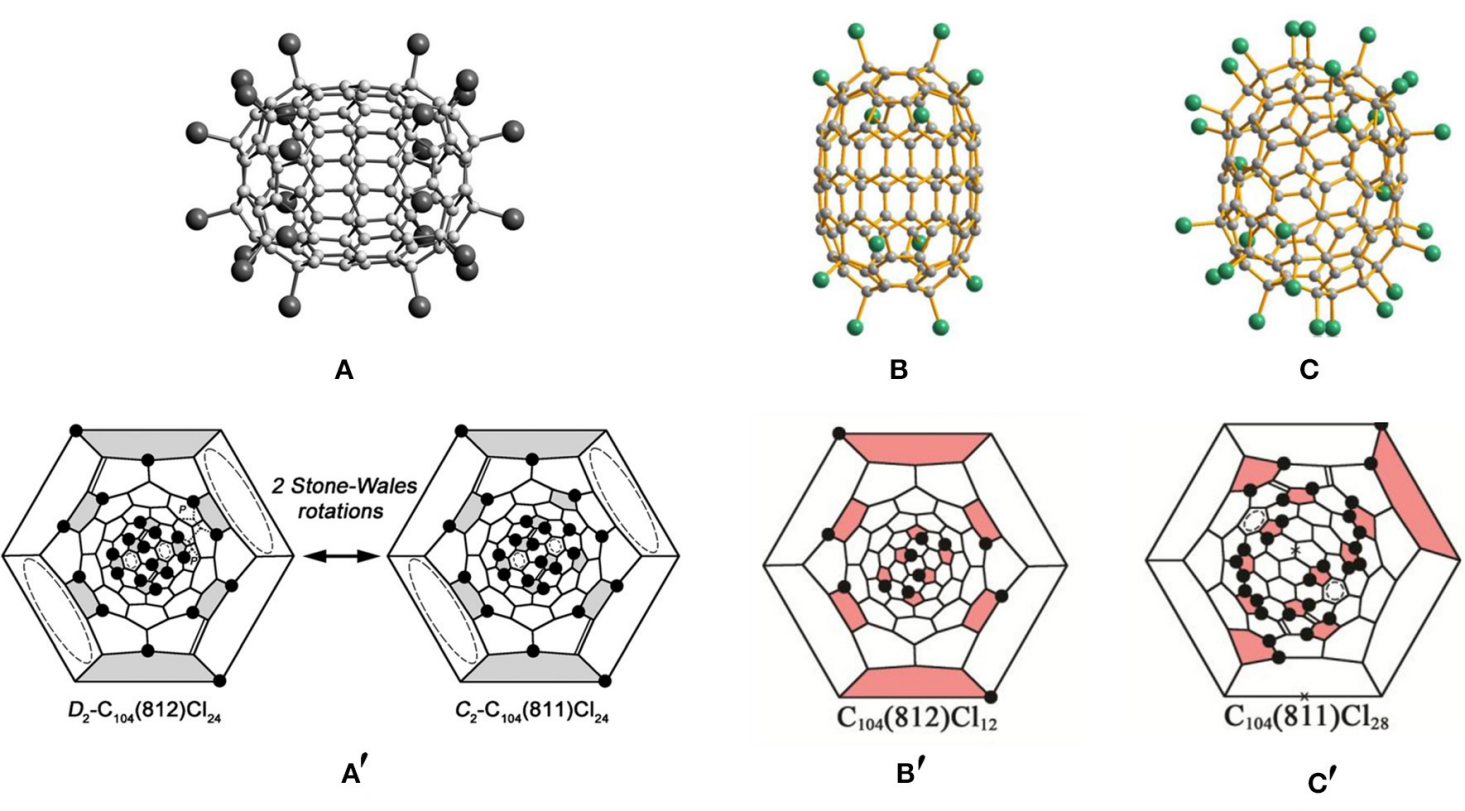
**(A)** Projections of the *D*_2_-C_104_(812)Cl_24_ molecule; **(A′)** Schlegel diagrams of *D*_2_-C_104_(812)Cl_24_ and *C*_2_-C_104_ (811)Cl_24_; **(B,B′)** projections and Schlegel diagrams of *D*_2_-C_104_(812)Cl_12_ and **(C,C′)** C_104_(811)Cl_28_.

The chlorination of the subfraction containing the giant fullerene *D*_2_-C_104_(812) first yields the chloride fullerene *D*_2_-C_104_(812)Cl_24_ (Figure 4A) (Yang et al., 2014c). As shown in Figure 4A′, 24 Cl atoms symmetrically are attached to the carbon cage, although the attached pattern of Cl atoms is non-uniform (Yang et al., 2014c). Each cage pentagon is occupied by two Cl atoms, and all of the Cl atoms are situated in the *para*-positions of the cage hexagons (Yang et al., 2014c). Therefore, four isolated benzenoid rings and four C=C double bonds have formed on the fullerene cage (Yang et al., 2014c). Notably, in the *D*_2_-C_104_(812)Cl_24_ molecule, there are two disordered C–C bonds formed by normal SWRs on one end of the cage, and their occupation ratio is 77:23 (Yang et al., 2014c). The alternative orientation of the disordered bonds corresponds to isomer C_104_(811). Hence, the structure should be regarded as a statistical overlap of the two isomers, *D*_2_-C_104_(812)Cl_24_ (major) and *C*_2_-C_104_(811)Cl_24_ (minor) (Yang et al., 2014c). Later, the C_104_(811) isomer has been solely captured as *C*_2_-C_104_(811)Cl_28_, but the attachment patterns of *C*_2_-C_104_(811)Cl_24_ and *C*_2_-C_104_(811)Cl_28_ are significantly different (see below) (Jin et al., 2017).

In 2017, two chloro-derivatives of *D*_2_-C_104_(812) with 12 and 24 Cl atoms attached were reported, and their molecular structures demonstrate crucial features of successive chlorination (Figure 4B) (Jin et al., 2017). Chlorination of *D*_2_-C_104_(812) takes place on two poles of the carbon cage alone, which retains its molecular symmetry. In detail, six Cl atoms uniformly attached to the six pentagons of each pole lead to the formation of an S-shaped *para*-chain. However, further propagation of chains on the ends is forbidden due to the presence of THJs in *para*-positions, as shown in Figure 4B′ (Jin et al., 2017). Similar kinds of limitations are also observed in the *D*_2_-C_84_(22)Cl_12_ molecule, which is quite understandable because of the close structural relationships between the *D*_2_-C_84_(22) and *D*_2_-C_104_(812) isomers: the inclusion of a belt of 20 carbon atoms between the two halves of *D*_2_-C_84_(22) produces *D*_2_-C_104_(812), both cages having the same symmetry and a very similar arrangement of six pentagons on each pole (Yang et al., 2014c). Moreover, the *D*_2_-C_104_(812)Cl_24_ molecule inherits the attachment features of the 12 Cl atoms of *D*_2_-C_104_(812)Cl_12_ (Jin et al., 2017). Additionally, there are 12 Cl atoms attached to the 1,3-positions of each pentagon (also to the *para*-position of the hexagon) in *D*_2_-C_104_(812)Cl_24_. However, this destabilizing structure is strengthened by producing four isolated C=C double bonds and four isolated benzenoid rings on the cage (Jin et al., 2017). As expected, DFT calculations demonstrate that the relative chlorination enthalpy of C_104_(812)Cl_24_ (2.5 kJ mol^−1^ per Cl) is much lower than that of C_104_(812)Cl_12_ (10.8 kJ mol^−1^) (Jin et al., 2017).

The structure of *C*_2_-C_104_(811)Cl_28_ is markedly different from that of *C*_2_-C_104_(811)Cl_24_, as shown in Figure 4C (Jin et al., 2017). Four isolated C=C double bonds and four benzenoid rings form in the carbon cage of *C*_2_-C_104_(811)Cl_24_, while the *C*_2_-C_104_(811)Cl_28_ molecule contains two nearly isolated benzenoid rings and four isolated C=C double bonds, two of the latter occurring in the same positions of *C*_2_-C_104_(811)Cl_24_ (Figure 4C′) (Jin et al., 2017). The most prominent difference observable in *C*_2_-C_104_(811)Cl_28_ is of the many Cl atoms attached at the *ortho*-positions on the cage involving two six-membered *ortho*-chains (Jin et al., 2017). However, the formation of relatively long *ortho*-chains is a typical characteristic of highly chlorinated fullerenes. The changes in the chlorination patterns of *C*_2_-C_104_(811)Cl_24_ with increasing degree of chlorination are quite similar to those of *T*_*h*_-C_60_Cl_24_/*C*_1_-C_60_Cl_28_; the chlorination pattern without any *ortho*-addition transforms into the structure with long *ortho*-chains with increasing degree of chlorination (Troyanov et al., 2005; Jin et al., 2017). Moreover, the relative chlorination enthalpy of C_104_(811)Cl_28_ (−0.1 kJ mol^−1^) is lower than that of C_104_(811)Cl_24_ (0.5 kJ mol^−1^) (Jin et al., 2017).

#### C_106_

**Figure 5:**
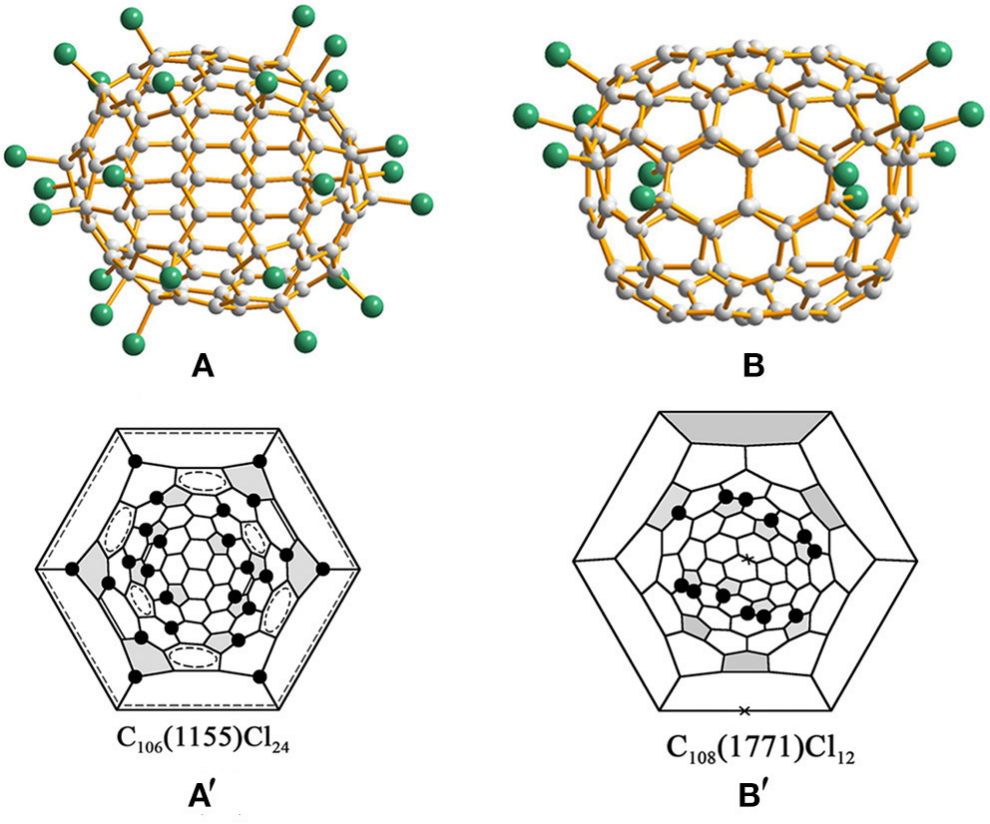
**(A,A′)** Projections and Schlegel diagrams of C_106_(1155)Cl_24_ and **(B,B′)** C_108_(1771)Cl_12_.

The structure of IPR C_106_(1155)Cl_24_ has been determined by chlorination of the giant fullerene C_106_, followed by a study using synchrotron radiation single-crystal X-ray diffraction (Figure 5A) (Wang et al., 2016b). Surprisingly, there are two molecules in the same crystal: one is C_106_(1155)Cl_24_ and the other is C_104_(*NC*)Cl_24_, with an *NC* carbon cage (Wang et al., 2016b). The occupancies of *C*_2_-C_106_(1155)Cl_24_ and *C*_1_-C_104_(*NC*)Cl_24_ are 23 and 77%, respectively, and the molecules show the same chlorination patterns and similar shapes (Wang et al., 2016b). Their inclusion in the same crystal packing is not hindered, and it is a common phenomenon of the co-crystallization of fullerene chlorides to have similar chlorination patterns but slightly different cages, e.g., C_78_(2,3)Cl_18_ (Simeonov et al., 2008) and C_90_(34,46)Cl_32_ (Kemnitz and Troyanov, 2009). As shown in Figure 5A′, the chlorination pattern of *C*_2_-C_106_(1155)Cl_24_ is characterized by the existing four isolated C=C double bonds and seven entirely isolated, or almost entirely isolated, benzenoid rings on the cage (Wang et al., 2016b). Furthermore, the presence of coronene and pyrene units on the poles of *C*_2_-C_106_(1155)Cl_24_ leads to the carbon cage being somewhat flattened (Wang et al., 2016b). Another unusual characteristic of the chlorination pattern is that four Cl atoms are attached to the THJs, which are generally unfavorable addition sites for fullerenes (Wang et al., 2016b). However, each addition at a THJ leads to the formation of two, or even three, benzenoid rings; thus, it is beneficial for stabilizing the molecule (Wang et al., 2016b).

#### C_108_

The chlorination reaction of the HPLC subfraction containing the giant fullerene C_108_ affords the fullerene chloride, *C*_2_-C_108_(1771)Cl_12_ (Figure 5B). Therefore, the presence of *D*_2_-C_108_(1771), the most stable isomer on the basis of theoretical calculations, has been confirmed in the fullerene soot (Wang et al., 2016b). As shown in Figure 5B′, the chlorination pattern of *C*_2_-C_108_(1771)Cl_12_ is characterized by 12 chlorine attachments non-uniformly distributed on the C_108_ cage. In detail, four pentagons are not occupied by Cl atoms, whereas half of the eight remaining pentagons bear two Cl atoms each (Wang et al., 2016b). This is different from the most stable addition pattern of the derivatives with 12 attached atoms or groups uniformly distributing on the carbon cage (Troyanov and Kemnitz, 2012). In general, the non-uniform attachments contribute to the formation of stabilizing substructures on the carbon cage, for example, benzenoid rings or isolated C=C double bonds (Troyanov and Kemnitz, 2012). However, there are no stabilizing substructures in *C*_2_-C_108_(1771)Cl_12_, although two separate areas of the cage contain both *para*- and *ortho*-additions of Cl atoms, as shown in Figure 5B′ (Wang et al., 2016b). In truth, two cage regions containing six pentagons are insulated by the extended region of the coronene units, which, as a result, prevents the generation of a single-addition chain (Troyanov et al., 2005). Depending on the theoretical calculations, further chlorination may occur at the positions on the second hemisphere of the *D*_2_-C_108_(1771) cage (Wang et al., 2016b).

### Heptagon-Containing Fullerenes Derived From Giant Fullerenes

#### C_96_(*NC*3)Cl_20_

**Figure 6:**
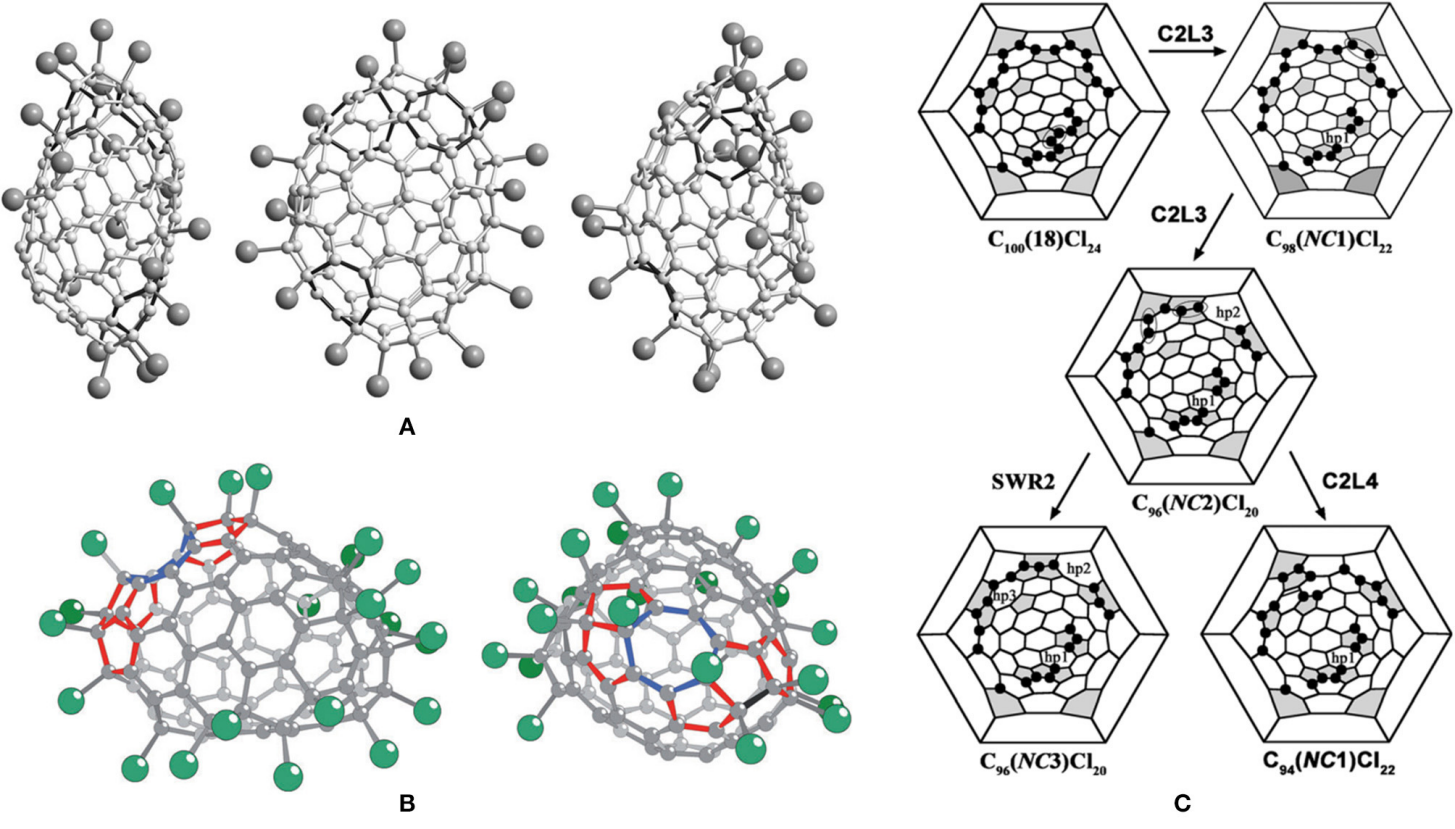
**(A)** Three projections of the *C*_1_-C_96_(*NC*3)Cl_20_ molecule; **(B)** two views of the *C*_1_-C_94_(*NC*1)Cl_22_ molecule; and **(C)** the shortest three-step pathways from a hypothetical IPR C_100_(18)Cl_24_ to the experimentally confirmed C_94_(*NC*1)Cl_22_ and C_96_(*NC*3)Cl_20_.

C_96_(*NC*3)Cl_20_ is a non-classical fullerene chloride, originating from the chlorination of C_100_(18), and, according to Euler's theorem, the carbon cage of C_96_(*NC*3)Cl_20_ has three heptagons and 15 pentagons (vs. 12 pentagons in classical fullerenes), as shown in Figure 6A (Yang et al., 2014a). In detail, there are three fused pentagon pairs formed, one sequentially fused triple, one directly fused triple, and three isolated pentagons in the cage (Yang et al., 2014a). Twenty Cl atoms are non-uniformly attached to the C_96_(*NC*3) cage, nine of them forming the chain of adjacent additions, the others forming shorter three- and four-membered chains (Figure 6C) (Yang et al., 2014a). As additional strain stems from the position of the fused pentagon in fullerene cages, all common edges of the fused pentagon pairs are chlorinated, which, remarkably, relieves the strain (Tan et al., 2009). In the sequentially fused triple of pentagons, three vertices of two common edges are chlorinated, which conforms to the rule drawn up previously for similar arrangements of pentagons (Tan et al., 2009). However, only three of the four vertices of fusion are chlorinated in the directly fused pentagon triple, which differs from the case of the non-IPR fullerene, C_64_Cl_4_ (Han et al., 2008), in which all four vertices of fusion are chlorinated (Yang et al., 2014a). In particular, two cage pentagons are not chlorinated, which has also been observed in higher fullerene chlorides with more than 12 attached groups, for example, C_104_(258)Cl_16_ (Yang et al., 2014a).

It is of great interest to seek the sources of the three heptagons, especially for the third heptagon. Apparently, two eliminations of 5:6 C–C bonds from the cage are responsible for the formation of the two heptagons, which is similar to the reported cases of IPR fullerene shrinkage contributing to the formation of the heptagonal rings (Troshin et al., 2005; Ioffe et al., 2010). However, the third heptagon is generated by an SWR of a 6:6 C–C bond, which joins a pentagon to a hexagon (Yang et al., 2014a). Such transformations are unprecedented for fullerenes, though an analogous rotation of a 6:6 bond in a pyrene-like fragment (four hexagons) is widely regarded as a mechanism for producing SW defects in nanotubes and graphenes (Dumitricǎ and Yakobson, 2004). Only three transformation steps (in any sequence) are necessary to reconstruct the probable pathway from C_100_ to C_96_(*NC*3), which involves two C_2_ losses and one SW rotation with the 6:6 type, as shown in Figure 6C (Yang et al., 2014a). Therefore, a possible three-step pathway has been proposed: C_2_ is lost as a chlorinated species (C_2_Cl_*n*_) occurring first, followed by the SW rotation (Yang et al., 2014a). The C_2_ loss and the heptagon generations are driven by simultaneously forming chlorinated sites at pentagon–pentagon adjacencies of fused pentagon pairs and within pentagon triples (Yang et al., 2014a). Obviously, the driving force for the formation of the third heptagon (hp3) produced by the SWR of a chlorinated C–C bond originates from producing additional chlorinated pentagon–pentagon junctions, especially the directly fused pentagon triple (Yang et al., 2014a). This transformation is strongly (103 kJ mol^−1^) exothermic on the basis of DFT calculations (Yang et al., 2014a). Moreover, one vertex of the directly fused pentagon triple remains unchlorinated due to no new chlorinated sites forming in the course of the SWR (Yang et al., 2014a).

#### C_94_(*NC*1)Cl_22_

In particular, non-classical C_94_(*NC*1)Cl_22_ containing one heptagon in the cage has been obtained together with the aforementioned C_96_(*NC*3)Cl_20_ having three heptagons from the same chlorination of *C*_2_-C_100_(18) (Ioffe et al., 2015). A concave region is formed in C_94_(*NC*1)Cl_22_ because the heptagons are flanked by a pair of fused pentagons and a sequential pentagon triple (Figure 6B) (Ioffe et al., 2015). As well, the C_94_(*NC*1) cage has two more pairs of fused pentagons and four isolated pentagons in the other areas of the carbon cage (Ioffe et al., 2015). Similarly, the 22 attached Cl atoms on the C_94_(*NC*1) cage are quite non-uniform, and several short *ortho*-chains dominate (Ioffe et al., 2015). Furthermore, the Cl atoms are attached mainly to the fused pentagons and the pentagon triple, and only one pentagon is unoccupied (Ioffe et al., 2015).

The shortest pathways from C_100_(18) to C_94_(*NC*1)Cl_22_ and C_96_(*NC*3)Cl_20_ involve two identical C2L steps, and branches at the hypothetically common precursor, C_96_(*NC*2)Cl_20_, have been put forward and are shown in Figure 6C (Ioffe et al., 2015). Notably, the carbon cage topologies of the missing starting and intermediate structures have been clearly established depending on the structural relations between the identified compounds, whereas the hypothetical aspect is their chlorination patterns, which are likely to occur as thermodynamicallydriven rearrangements, the so-called “chlorine dance.” (Ioffe et al., 2015). A new kind of C_2_ loss exclusive to non-IPR compounds has been proposed: C2L4 means removing the 5:5 C–C bond from a pentalene fragment, in other words, from a fused pair of pentagons (Ioffe et al., 2015). As a consequence, the pentalene unit transforms into a hexagon, whereas the adjacent hexagon and heptagon are reduced to a pentagon and a hexagon (Ioffe et al., 2015). Hence, C_94_(*NC*1)Cl_22_ containing only one heptagon has been achieved, as a result of C2L4 reverting a non-classical fullerene to a classical carbon cage (Ioffe et al., 2015). Additionally, both the SWR2 and C2L4 processes have been studied using DFT calculations in order to estimate which one is preferable (Ioffe et al., 2015). The process of SWR2 has a sizeable exothermic effect of 105 kJ mol^−1^ and an activation barrier of 180 kJ mol^−1^, which are among the lowest values previously calculated for such processes (Ioffe et al., 2015). The C2L4 elimination mechanism suggested by DFT contains complex intermediate and transient states (Ioffe et al., 2015). Finally, the calculated activation energies SWR2 and C2L4 are comparative, and the latter possesses slightly lower energy barriers (Ioffe et al., 2015). In addition, the computational results simultaneously substantiate the aforementioned shortest pathways with competitive transformations of the hypothetical common precursor, C_96_(*NC*2)Cl_20_ (Ioffe et al., 2015).

#### C_98_(*NC*1)Cl_26_

**Figure 7:**
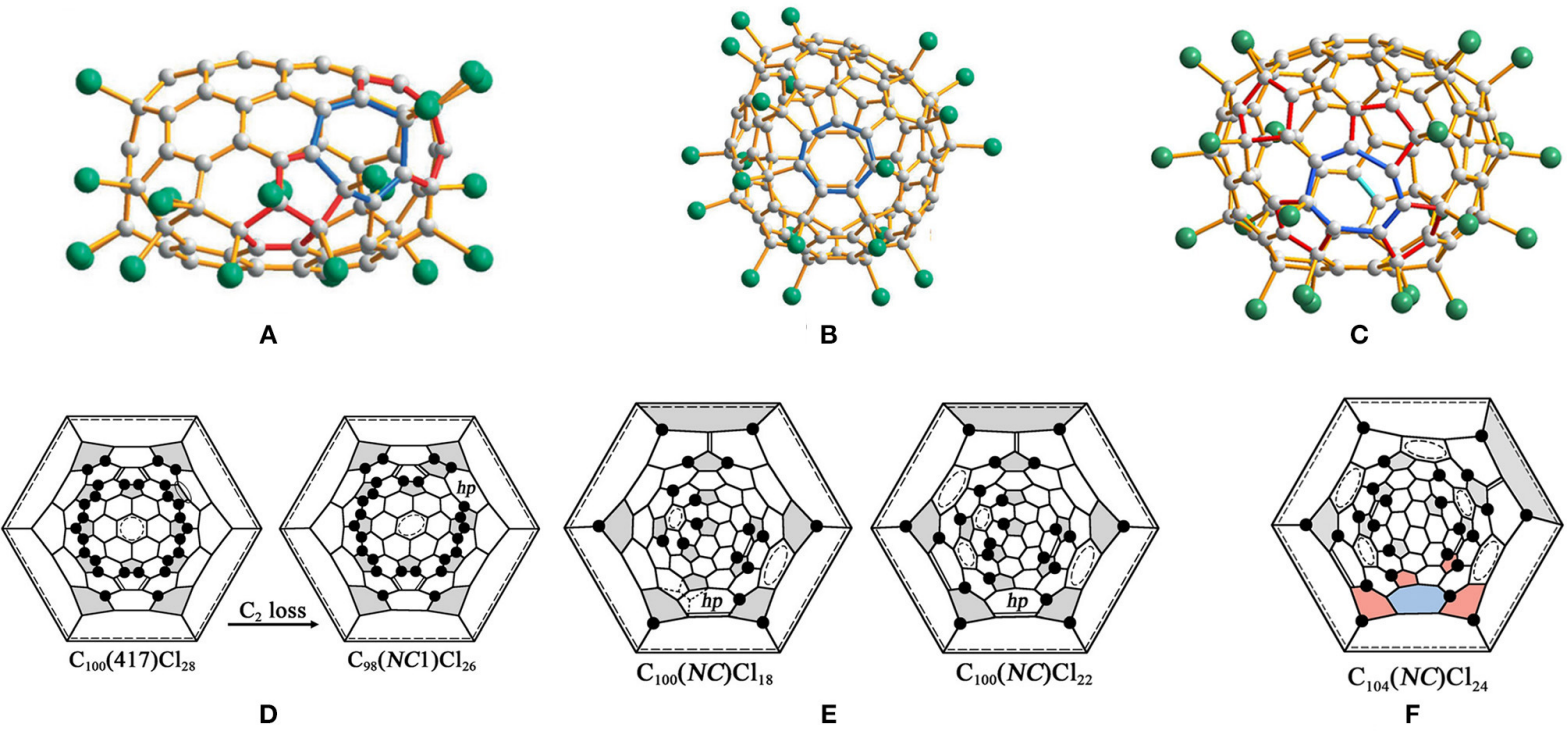
Projections of molecules: **(A)** C_98_(*NC*1)Cl_26_; **(B)** C_100_(*NC*1)Cl_22_; and **(C)** C_104_(*NC*1)Cl_24_; **(D)** Schlegel diagrams of *C*_*s*_-C_100_(417)Cl_28_ and *C*_1_-C_98_(*NC*1)Cl_26_; **(E)** Schlegel diagrams of C_100_(*NC*1)Cl_18/22_ and **(F)** C_104_(*NC*1)Cl_24_.

Non-classical C_98_(*NC*1)Cl_26_ with one heptagon rooted in another isomer of C_100_, C_100_(417), forms a co-crystal with C_100_(417)Cl_28_ at the same crystallographic site (Figure 7A), and their occupancies are 0.529 and 0.471, respectively (Wang et al., 2016a). Comparative analysis of the two structures indicates that a heptagon of the non-classical C_98_(*NC*1)Cl_26_ originates from C_100_(417)Cl_28_ via the C_2_ loss of a 5:6 C–C bond (Figure 7D) (Wang et al., 2016a). In fullerenes, this type of C_2_ loss is designated as C2L2 on the basis of the topological classification of skeletal transformations, which is similar to the transformation observed in isomers of C_96_ fullerene (Yang et al., 2014d). Significantly, the stability of the resulting C_98_(*NC*1)Cl_26_ has been strengthened by Cl atom attachments to two pentagon–pentagon fusions (Wang et al., 2016a).

#### C_100_(*NC*1)Cl_18/22_

Both of the chlorinated derivatives, *C*_1_-C_100_(*NC*1)Cl_18_ (Figure 7B) and *C*_1_-C_100_(*NC*1)Cl_22_, have been confirmed in the same crystal, which contains the same non-classical C_100_ cage with one heptagon (Wang et al., 2016a). In addition, 17 Cl atom attachments, including one THJ of a chlorination pattern, occur in both structures (Wang et al., 2016a). The carbon cage of C_100_(*NC*1)Cl_18_ contains three isolated and nearly isolated C=C double bonds and three isolated and nearly isolated benzenoid rings, whereas the corresponding numbers for C_100_(*NC*1)Cl_22_ are four and five, as shown in Figure 7E (Wang et al., 2016a). In particular, the most important question regarding these structures relates to the origins of the non-classical C_100_(*NC*1) cage of the two characterized chloro-derivatives (Wang et al., 2016a). No fullerene beyond C_100_ in the starting fullerenes was observed; therefore, a transformation from even higher fullerenes to targeted non-classical fullerenes by a common C_2_ loss should be excluded (Wang et al., 2016a). Furthermore, C_100_(*NC*1) can be obtained by a single SW rearrangement of the type SWR2 from the IPR isomers C_100_(382) or C_100_(344). However, isomer C_100_(382) has been considered as the starting fullerene because of its relatively lower formation energy compared with C_100_(344). But there is no obvious driving force from IPR C_100_(382) or C_100_(344) to C_100_(*NC*) due to the final cage not containing fused pentagons (Wang et al., 2016a). Therefore, an alternative option is that the non-classical C_100_ (*NC*), having a comparable low formation energy, may exist in the starting fullerene used for chlorination (Wang et al., 2016a).

#### C_98_(*NC*2)Cl_26_

**Figure 8:**
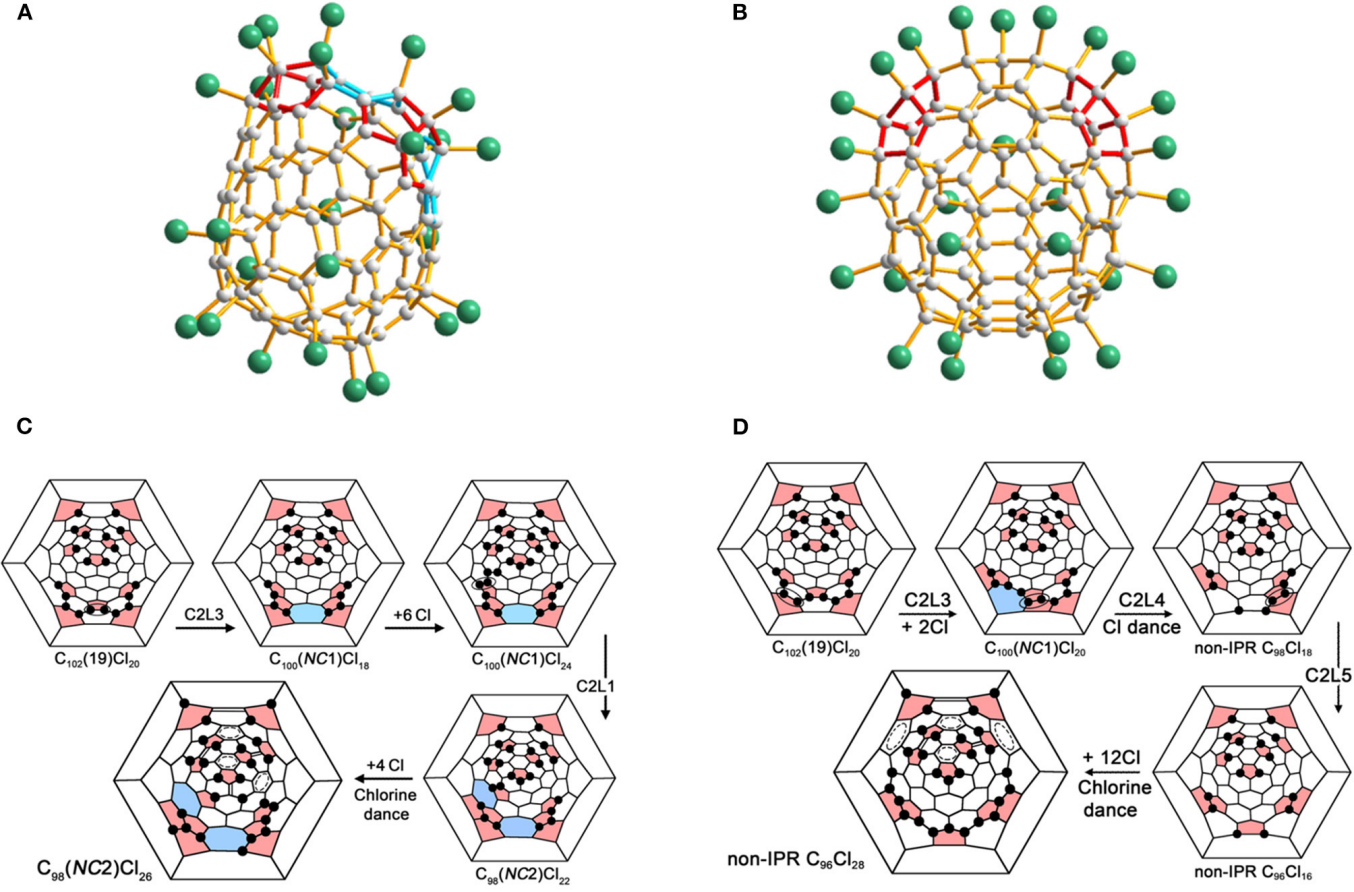
**(A)** Views of C_98_(*NC*2)Cl_26_ and **(B)** non-IPR C_96_Cl_28_; **(C)** Schlegel diagram presentations of the pathways from C_102_(19) to the non-classical C_98_(*NC*2)Cl_26_ via two C2L steps; **(D)** from C_102_(19) to the non-classical C_96_Cl_28_ via three C2L steps.

Non-classical C_98_(*NC*2)Cl_26_ containing two heptagons has been synthesized from C_102_(19) via two C2L steps without any accompanying SWR processes (Figure 8A) (Mazaleva et al., 2018). A C2L step and an additional chlorination step take place alternately, and the so-called “chlorine dance,” equilibrium rearrangement of chlorination patterns, is also involved in the last step of the additional chlorination (Mazaleva et al., 2018). As shown in Figure 8C, the designations C2L1 and C2L3 relate to removing the pentagon–hexagon edge where the hexagon has one or three adjacent pentagons (Mazaleva et al., 2018). Remarkably, the C2L and SWR processes within the parent ^#283794^C_102_Cl_20_ occur in the same area of the carbon cage, and the probable reason is that the chlorination pattern of the parent C_102_(19) is characterized by a successive chain of adjacent chlorine attachments in that area (Mazaleva et al., 2018). Unexpectedly, novel non-IPR C_96_Cl_28_ was captured by chlorination of IPR ^#341061^C_102_ under the same conditions of just prolonging the reaction time (Figure 8B) (Yang et al., 2013; Mazaleva et al., 2018). As shown in Figure 8D, the formation process of the non-IPR C_96_Cl_28_ (or ^#185115^C_96_Cl_28_) has three C2L steps and two pathways, as the order of the initial steps is an alternative, and one of them is the same as in the case of C_98_(*NC*2)Cl_26_ (Mazaleva et al., 2018). One of the common pentagon–pentagon edges is eliminated in the second step, which destroys the heptagon formed in the previous step, in both cases (Mazaleva et al., 2018). Dramatically, the third step represents a novel C2L5 process, which eliminates a common pentagon–pentagon edges surrounded by two hexagons, and thus neither creates nor destroys any heptagons (Mazaleva et al., 2018). The comparable activation energies of C_98_(*NC*2)Cl_26_ and non-IPR C_96_Cl_28_ provided by the DFT calculations lead to the concurrent formation of two derivatives under the same conditions (Mazaleva et al., 2018).

#### C_104_(*NC*1)Cl_24_

The non-classical chloride *C*_1_-C_104_(*NC*1)Cl_24_ with one heptagon in the carbon cage forms a co-crystal with *C*_2_-C_106_(1155)Cl_24_ (Figure 7C) (Wang et al., 2016b). The molecular structure of *C*_1_-C_104_(*NC*1)Cl_24_ differs from that of *C*_2_-C_106_(1155)Cl_24_ by a rotated C–C bond in one cage region along with the presence of a heptagon in another cage region (Wang et al., 2016b). As a consequence, six benzenoid rings and three isolated C=C double bonds form on the carbon cage; the former is one less than that in *C*_2_-C_106_(1155)Cl_24_, whereas the latter is equal to that in *C*_2_-C_106_(1155)Cl_24_ (Wang et al., 2016b). The addition positions of the 24 Cl atoms are similar to those in *C*_2_-C_106_(1155)Cl_24_, while two Cl atoms attach in the THJs, as shown in Figure 7F (Wang et al., 2016b). C_106_(1158) could be regarded as the starting fullerene, with a relative formation energy of 38 kJ mol^−1^ (Wang et al., 2016b). However, this assumption is doubtful because no fused pentagons around the heptagon have been found, which is typically observed for the previous case of the C_2_ loss from fullerene cages (Wang et al., 2016b). Alternatively, isomer C_104_(*NC*) with one heptagon and 13 pentagons (but no fused pentagons) is also a candidate present in the fullerene soot (Wang et al., 2016b). In particular, the relative formation energy of C_104_(*NC*) is only 40 kJ mol^−1^ higher than that of the most stable IPR isomer C_104_(234), whereas it is even lower than those of the experimentally confirmed C_104_(811) and C_104_(258), which have relative formation energies of 44 and 57 kJ mol^−1^, respectively (Wang et al., 2016b).

### Fullertubes

**Figure 9:**
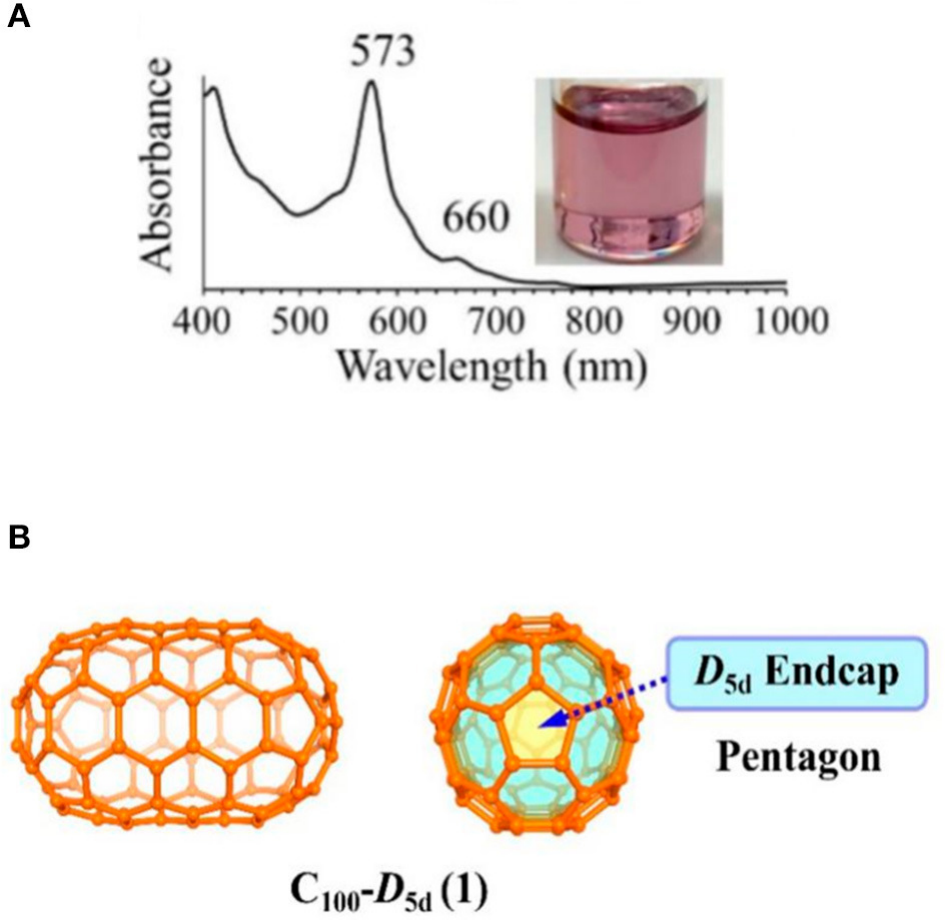
**(A)** The UV-vis spectrum of fullertube *D*_5*d*_-C_100_(1), **(B)** schematic diagram of fullertube *D*_5*d*_-C_100_(1).

Very recently, Koenig et al. reported fullertubes possess single-walled carbon nanotube belts resembling a rolled graphene midsection, but with half-fullerene end-caps (Koenig et al., 2020). Fullertubes were isolated by a chemical method, which spheroidal fullerene cages highly reacted with the amines and were removing out. Then the remaining fullertubes were subjected by a simple HPLC separation and achieved purified fullertubes. *D*_3*d*_-C_96_(3), *D*_5*h*_-C_90_(1), and *D*_5*d*_-C_100_(1) fullertubes were obtained when the toluene was used as the solvent (Koenig et al., 2020). The fullertubes were characterized in pristine and unfunctionalized form by UV-Vis spectra (Figure 9A) and single crystal X-ray diffraction. The UV-vis spectrum of the isolated C_96_ matches that reported in the literature confirming by single crystal X-ray diffraction, so the isolated C_96_ fullertubes is assigned to *D*_3*d*_-C_96_(3) with hexagon end-caps (Koenig et al., 2020). Furthermore, the results of single crystal X-ray diffraction, clearly show that both structures of *D*_5*d*_-C_100_(1) and *D*_5*h*_-C_90_(1) fall into the set of fullertube structures with pentagon poles (Figure 9B) and general formula of C_30+30+10n_ with *D*_5*h*_ (if n is odd, C_90_) or *D*_5*d*_ symmetry (if n is even, C_100_) (Koenig et al., 2020). Furthermore, giant fullertubes, such as C_108_, C_120_, C_132_, and C_156_ were obtained when toluene was replaced by xylene dissolving the as-generated carbon soot. By overlaying the mathematical series of fullertubes with the mass spectral data, the isolated C_120_ may likely be tubular. But the possibility of chemically stable spheroidal shape of C_120_ can not be ruled out, because two structural isomers possibly exist due to the noticeably broad HPLC peak for C_120_. Moreover, the isolated C_108_, C_132_, and C_156_ might correspond to these predicted spiral fullertubes on the basis of Mathematical and mass spectrum analysis (Koenig et al., 2020).

## Giant Endohedral Fullerenes

### Isomer Structures and Endohedral Species

#### M_2_@C_2*n*_(2*n*≥100)

Dy_2_@C_100_ was the first giant endohedral metallofullerene experimentally characterized by various spectral methods in 2006 (Yang and Dunsch, 2006). Based on the absorption spectral onset of 1,590 nm, the optical band gap is calculated to be 0.78 eV, which demonstrates that Dy_2_@C_100_ is a small band gap fullerene (Yang and Dunsch, 2006). Furthermore, Dy_2_@C_100_ exhibits instability in the solid form, which is confirmed by the existence of strong, but unresolved, absorption bands between 870 and 1,260 cm^−1^ in the Fourier transform infrared (FTIR) spectra (Yang and Dunsch, 2006). Such broad bands are attributed to the graphitization of the dimetallofullerenes (Krause et al., 2001). Isomer 449:D_2_ was calculated to be the lowest-energy isomer of C_100_ (Yang and Dunsch, 2006). However, five isomers, 18:*C*_2_, 426:*C*_1_, 425:*C*_1_, 442:*C*_2_, and 148:*C*_1_, are preferentially populated within a wide temperature interval according to the DFT calculations (Yang and Dunsch, 2006). All of the aforementioned six thermodynamically most stable isomers are regarded as the probable cage candidates for Dy_2_@C_100_ because fullerenes are synthesized at extremely high temperatures by arc discharges (Yang and Dunsch, 2006).

**Figure 10:**
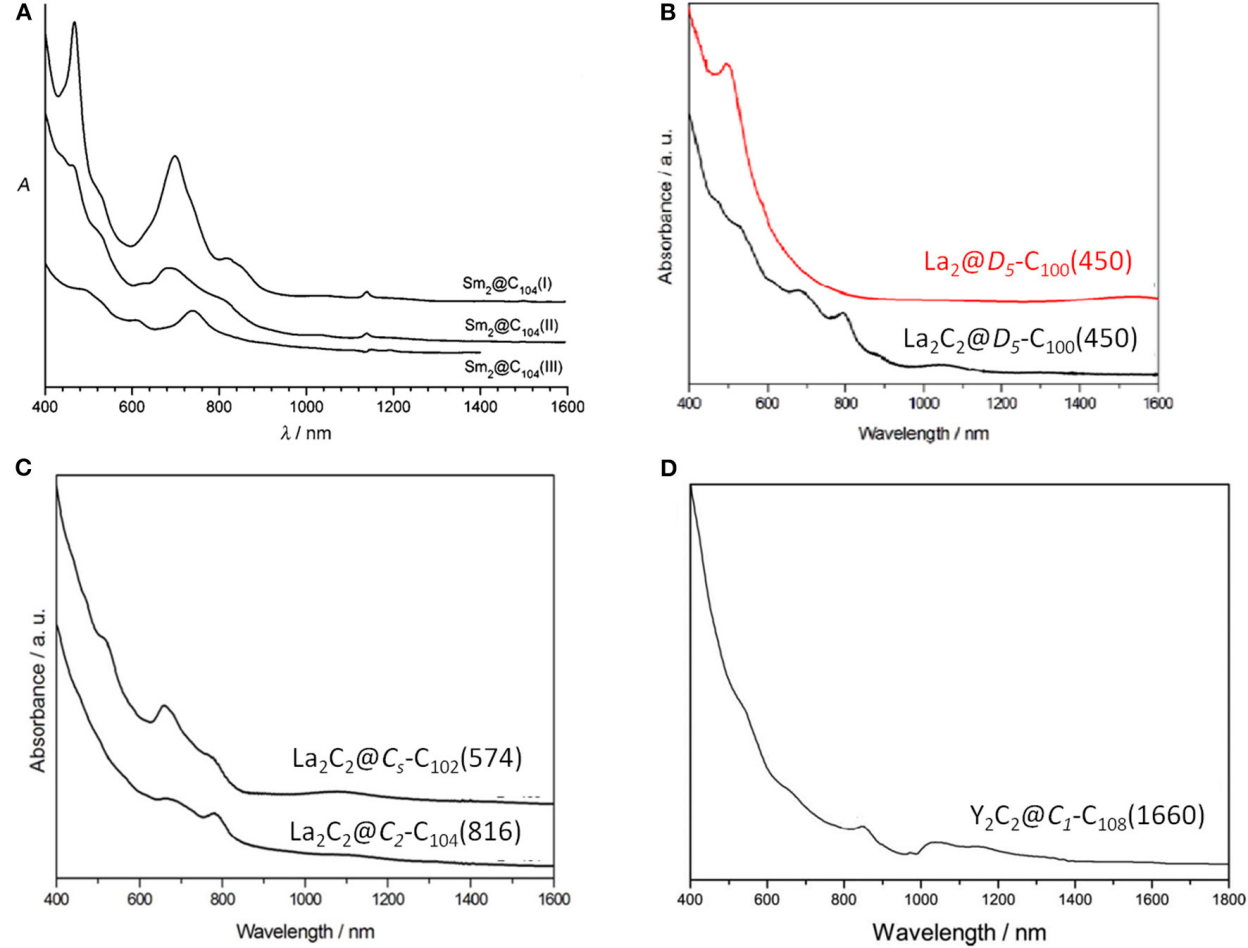
Vis-NIR absorption spectra of **(A)** Sm_2_@C_104_(I,II,III); **(B)** La_2_@*D*_5_-C_100_(450) and La_2_C_2_@*D*_5_(450)-C_100_; **(C)** La_2_C_2_@*C*_*s*_-C_102_(574) and La_2_C_2_@C_2_-C_104_(816); and **(D)** Y_2_C_2_@*C*_1_-C_108_(1660).

**Figure 11:**
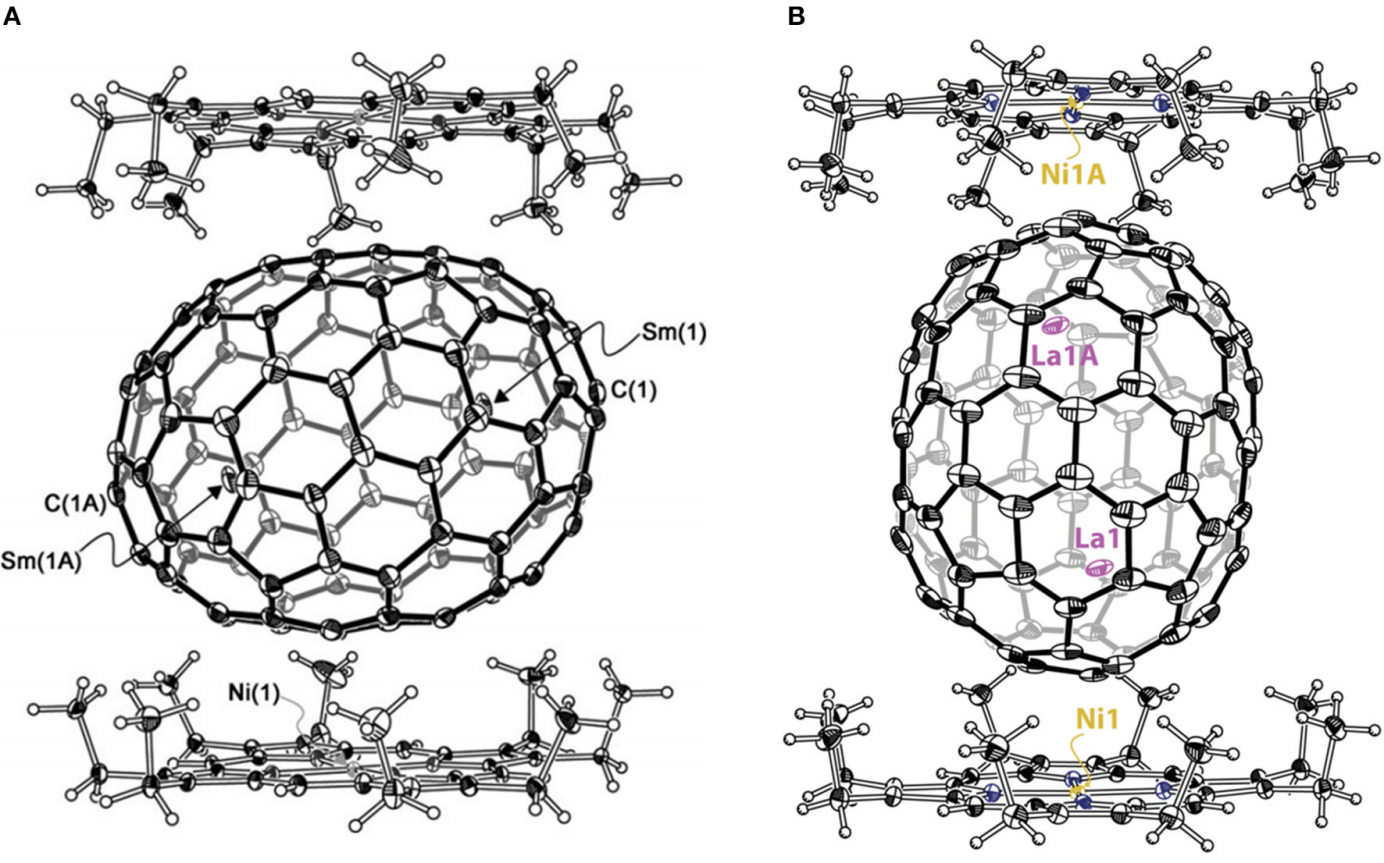
**(A)** Sm_2_@*D*_3*d*_-C_104_(822) and the two surrounding Ni^II^(OEP) molecules. Two primary Sm atoms (with 0.74 fractional occupancy) are shown; **(B)** La_2_@*D*_5_-C_100_(450) and two molecules of Ni (OEP) are shown. Only one orientation of the C_100_ cage and the major lanthanum (La) site along with the symmetry-generated La1A site are shown.

Sm_2_@*D*_3*d*_-C_104_(822) is the first giant EMF to be unambiguously confirmed by single-crystal X-ray diffraction (Mercado et al., 2009). Three individual isomers of Sm_2_@C_104_ were isolated and purified, and their UV/Vis/NIR absorption spectra are presented in Figure 10A. The first eluted isomer I, with nickel octaethylporphyrin Ni(OEP) formed a black co-crystal (Mercado et al., 2009). The asymmetric unit of the crystal is made up of one molecule of Ni(OEP), one-half of the fullerene with the other half produced by a center of symmetry, and one-half of a disordered chlorobenzene molecule (Mercado et al., 2009). The crystallographic data demonstrate Sm_2_@C_104_(I) to be a conventional endohedral fullerene (Figure 11A) and not a carbide fullerene (Mercado et al., 2009). Sm_2_@C_104_(I) has a carbon cage of *D*_3*d*_-C_104_(822), which is the only one of the 823 isomers of C_104_ obeying the IPR to possess *D*_3*d*_ symmetry (Mercado et al., 2009). In detail, samarium atoms show three disorder defects, and the occupancy of the major site is 0.74 and those of the two nearby sites are 0.17 and 0.09 (Mercado et al., 2009). Two primary Sm atoms are situated near the 3-fold axis of the carbon cage at a distance of 5.8322(7) Å in the molecule (Mercado et al., 2009). Each Sm atom is located beneath a canopy of three adjacent hexagonal rings, and the shortest Sm–C distance is 2.521(5) Å (Mercado et al., 2009). This cage is elongated, and its length, as measured by the distance between C1 and C1A lying on the 3-fold axis, is 10.840(9) Å, whereas its diameter is 8.264(9) Å (Mercado et al., 2009). Furthermore, the cage is closely related to a capped armchair carbon nanotube as well as to the structures of the *I*_*h*_ and *D*_5*h*_ isomers of C_80_ (Mercado et al., 2009). Specifically, the *D*_3*d*_-C_104_(822) cage is generated by addition of 24 carbon atoms to fragments produced by the *I*_*h*_-C_80_ cage cutting perpendicular to the *C*_3_ axis (Mercado et al., 2009). In addition, the electronic distribution of Sm_2_@*D*_3*d*_-C_104_(822) is (Sm^2+^)_2_@[*D*_3*d*_-C_104_^4-^(822)] suggested by the computational data (Mercado et al., 2009).

La_2_@*D*_5_-C_100_(450) was isolated by extensive chromatographic separations, though a series of giant endohedral metallofullerenes from La_2_C_90_ to La_2_C_138_ were confirmed by MS (Beavers et al., 2011). The UV/Vis/NIR spectra of La_2_@*D*_5_-C_100_(450) shown in Figure 10B are different from those of Dy_2_C_100_ having a low-energy absorption band at ~1,060 nm (Yang and Dunsch, 2006; Beavers et al., 2011). It is possible that different cages for the two compounds or paramagnetic dysprosium (Dy) may lead to this discrepancy (Beavers et al., 2011). In the co-crystal, La_2_@*D*_5_-C_100_(450) is immobilized by two Ni(OEP) molecules, one at each end (Figure 11B) (Beavers et al., 2011). The carbon cage is chiral but occupies a centrosymmetric site in the crystal (Beavers et al., 2011). Both the carbon cage and the lanthanum ions suffer from disorders, specifically, four nearly populated sites for the carbon cage, two for each enantiomer, and four sites for the La ions. The occupancies of the La ions are 0.6891(13), 0.1242(16), 0.1072(10), and 0.0794(14), respectively (Beavers et al., 2011). La_2_@*D*_5_-C_100_(450) is a conventional dimetallofullerene and not a carbide fullerene, as is the Sm_2_@*D*_3*d*_-C_104_(822) (Beavers et al., 2011). The nanotubular shape of La_2_@*D*_5_-C_100_(450) resembles those of Sm_2_@*D*_3*d*_-C_104_(822) (Mercado et al., 2009) and *D*_5*h*_-C_90_(1) (Yang et al., 2010). The centroid-to-centroid distance between pentagons on the major axis is 10.083 Å, while five perpendicular 2-fold axes bisecting the 6:6 ring junctions are being, and their average centroid-to-centroid distance is 8.024 Å (Beavers et al., 2011). The cage structure is *D*_5_-C_100_(450) according to theoretical predictions, which is most appropriate for encapsulating the (M^3+^)_2_ unit (Beavers et al., 2011). This cage also satisfies the maximal pentagon separation rule: the physics of fullerene stabilization by requiring maximal separation between the 12 pentagons (Rodríguez-Fortea et al., 2010). The La ions can be observed to reside in the curved poles of the cage located by the pentagons (Beavers et al., 2011). Simultaneously, two La ions diverge slightly from the fivefold axis of the carbon cage and are widely separated by a distance of 5.7441(4) due to the repulsion of the two cations, which is similar to the cases of other La-containing endohedrals (Beavers et al., 2011). Additionally, in the crystal, the long axis of the La_2_@*D*_5_-C_100_(450) molecule is perpendicular to the planes of the two porphyrins (Beavers et al., 2011). Hence, the most curved part of the carbon cage is close to the planar Ni(OEP) molecules. In contrast, the less-curved interior portions of Sm_2_@*D*_3*d*_-C_104_(822) are adjacent to the two neighboring Ni(OEP) molecules (Beavers et al., 2011). As a result, in La_2_@*D*_5_-C_100_(450)·2Ni(OEP)·2(toluene), the Ni1—Ni1A separation of 15.8785(6) Å across the carbon cage is longer than the corresponding Ni—Ni separation of 14.3850(13) in the centrosymmetric Sm_2_@*D*_3*d*_-C_104_(822)·2Ni(OEP) C_6_H_5_Cl (Beavers et al., 2011).

#### M_2_C_2_@C_2*n*_(2*n*≥100)

**Figure 12:**
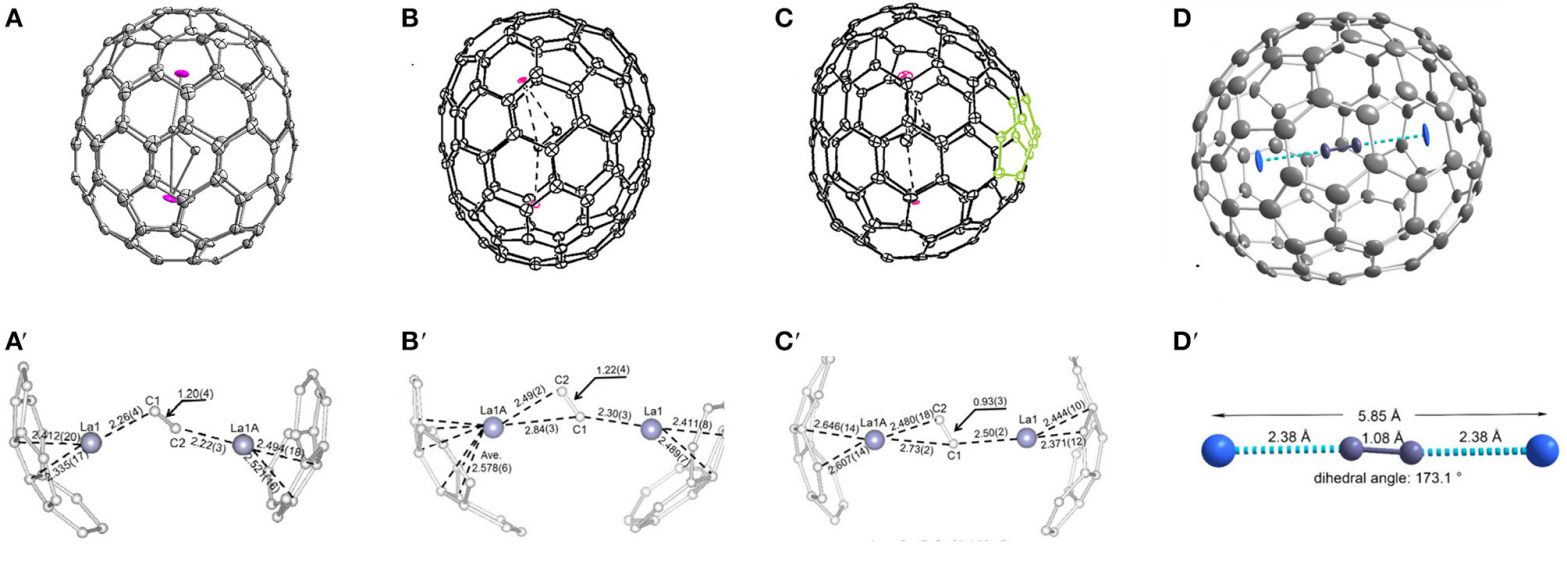
**(A)** schematic diagram of the cage of La_2_C_2_@*D*_5_-C_100_(450); **(A′)** distribution of La positions beneath the two poles of the cage; **(B)** ORTEP drawings of La_2_C_2_@*C*_*s*_-C_102_(574); **(B′)** positions of the major La_2_C_2_ cluster relative to a partial cage of La_2_C_2_@*C*_*s*_-C_102_(574); **(C)** ORTEP drawings of La_2_C_2_@*C*_2_-C_104_(816); **(C′)** positions of the major La_2_C_2_ cluster relative to a partial cage of La_2_C_2_@*C*_2_-C_104_(816); **(D)** view of the Y_2_C_2_@*C*_1_-C_108_(1660) molecule; **(D′)** structural parameters of the Y_2_C_2_ cluster.

La_2_C_2_@*D*_5_-C_100_(450) was unambiguously confirmed as a carbide fullerene by single-crystal X-ray diffraction (Figure 12A) (Cai et al., 2015). The cage isomer, *D*_5_-C_100_(450), is the same as that of La_2_@*D*_5_-C_100_(450). However, the Vis-NIR spectrum is significantly different from those of La_2_@*D*_5_-C_100_(450) and Dy_2_@C_100_ (Figure 10B), which indicates that their electronic configurations differ (Cai et al., 2015). Unexpectedly, in the co-crystal, the Ni(OEP) added as a co-crystallization host is absent, leaving only the fullerene and the intercalated CS_2_ molecules (Cai et al., 2015). Both the carbon cage and the embedded La_2_C_2_ cluster show several disorder defects, and the chiral fullerene cage has two disordered enantiomers with almost equal occupancy (0.52:0.48) (Cai et al., 2015). There are 19 sites for two La ions, and the major two sites are over a respective [6,6]-bond junction near a pole of the cage passing the fivefold axis of the cage (Figure 12A′) (Cai et al., 2015). The inner C_2_ unit possesses four disordered sites with C–C bond distances of 1.00–1.21 Å (Cai et al., 2015). Furthermore, numerous disordered sites with La ions and C_2_ units demonstrate the free movement of metal atoms and the flexible swing of the C_2_ unit within the carbon cage (Cai et al., 2015). The La–La separation distance of the major sites is 4.83 Å, which is obviously shorter than that of La_2_@*D*_5_-C_100_(450) (Cai et al., 2015). The La_2_C_2_ cluster shows a bent configuration, with a dihedral angle of 141.3° between the two LaC_2_ portions (Cai et al., 2015). Moreover, the C_2_ unit is considered to be rotating in the cluster plane, which confirms the computed prediction of the possibility of the linear M_2_C_2_ cluster structures in the giant fullerene (Cai et al., 2015).

**Figure 13:**
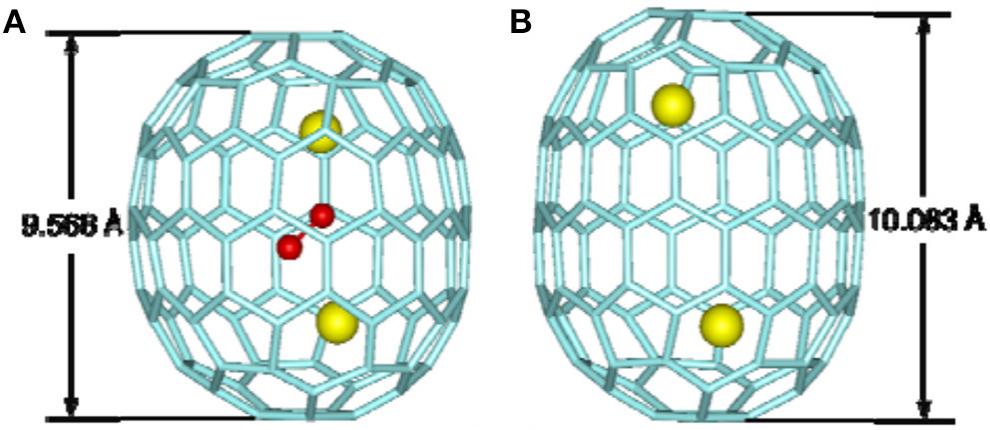
X-ray structures of: **(A)** La_2_C_2_@*D*_5_-C_100_(450) and **(B)** La_2_@*D*_5_-C_100_(450).

Most notably, the anomalous axial compression of *D*_5_-C_100_(450) is clearly observed when the structures of La_2_C_2_@*D*_5_-C_100_(450) and La_2_@*D*_5_-C_100_(450) are compared (Figure 13) (Cai et al., 2015). The length of the cage in La_2_@*D*_5_-C_100_(450) is 10.083 Å, and the width of the cage is 8.024 Å. In contrast, the long axis of La_2_C_2_@*D*_5_-C_100_(450) reduces to 9.585 Å, but the width of the cage is 8.332 Å, that is, slightly expanded (Cai et al., 2015). This result clearly reveals the larger cluster La_2_C_2_ obviously contracts the carbon cage, rather than expanding it (Cai et al., 2015). Moreover, the La–La separation distance of the two major La atoms in La_2_C_2_@*D*_5_-C_100_(450) (4.830 Å) is apparently shorter than that in La_2_@*D*_5_-C_100_(450) (5.744 Å), while the La–cage distances are nearly equal in the two molecules (Cai et al., 2015). The reason for the shortened La–La distance is that the positive charge is partly neutralized by the electronegative C_2_ unit and the Coulombic repulsion between the two La ions is weakened (Cai et al., 2015). Hence, the axial compression of the carbon cage may result from the stronger bonding interactions between the La ions and the C_2_ unit (Cai et al., 2015). Based on the calculated X-ray results for La_2_C_2_@*D*_5_-C_100_(450) and La_2_@*D*_5_-C_100_(450), the whole axial strain of this small capped zigzag (10,0) nanotube, *D*_5_-C_100_(450), is 5% (Cai et al., 2015). Detailed analyses reveal that the [10] cyclacene sidewall segment containing purely [6,6]-bonds is responsible for the structural deformation, but that the pentagon-dominating corannulene caps are very rigid (Cai et al., 2015).

La_2_C_2_@*C*_*s*_-C_102_(574) was also isolated and characterized by HPLC, Vis-NIR spectra, and single-crystal X-ray diffraction, as shown in Figure 12B (Cai et al., 2016). Its Vis-NIR spectrum shown in Figure 10C indicates that it has a small HOMO-LUMO gap due to the spectral onset at around 1,300 nm, which is similar to those reported for the giant endohedral metallofullerenes (Cai et al., 2016). Similarly, the crystal units contain merely one La_2_C_2_@*C*_*s*_-C_102_(574) molecule and two CS2 molecules, whereas the co-crystallization host Ni (OEP) used, as well as other solvent molecules, are absent (Cai et al., 2016). There are two bands of 10 contiguous hexagons encircling the cage, which is similar to the previously reported tubular *D*_3*d*_-C_104_(822), *D*_5_-C_100_(450) and *D*_5*h*_-C_90_(1). Within the cage, the carbide cluster shows several disordered positions, and there are 18 La positions for the two La atoms, which display as an umbrella shape relative to the three disordered sites of the C_2_ unit (Cai et al., 2016). Furthermore, the two major La ions in La_2_C_2_@*C*_*s*_-C_102_(574) are detached and the line connecting them is a little displaced from the long axis of the carbon cage (Cai et al., 2016). One of them is situated under a hexagon, while the other is located over a [5,6]-bond on the opposite side (Figure 12B′) (Cai et al., 2016). The La_2_C_2_ unit shows a stretched and nearly planar configuration, which differs from the bent butterfly-like configuration dominating in the Sc_2_C_2_ cluster fullerene (Kurihara et al., 2012). Moreover, the disordered C_2_ unit is no more perpendicular to the line crossing the two major La ions (Cai et al., 2016). The La–C–C–La dihedral angle (173.6°) in La_2_C_2_@*C*_*s*_-C_102_(574) is much larger than that in La_2_C_2_@*D*_5_-C_100_(450) (141.3°) (Cai et al., 2016). This demonstrates that the carbide cluster transforms from a slightly bent structure into a nearly planar configuration as the cage length increases, which is consistent with the theoretical predictions that the M_2_C_2_ cluster may prefer a linear geometry in large cages (Zhang et al., 2012).

La_2_C_2_@C_2_-C_104_(816) is also unambiguously confirmed as a carbide by single-crystal X-ray diffraction, as shown in Figure 12C (Cai et al., 2016). The spectral onset of the Vis-NIR spectrum occurs at ~1,300 nm (Figure 10C), resulting in a small HOMO-LUMO gap (Cai et al., 2016). The analogous crystallization behavior as described above is also present, where the co-crystallization host Ni(OEP) used is absent from the co-crystal (Cai et al., 2016). The cage of La_2_C_2_@*C*_2_-C_104_(816) shows a “defective” tubular structure resulting from the insertion of a pyracylene unit into the two bands of hexagons on the waist of the cage and leading to a reduction in the symmetry of the cage (Cai et al., 2016). Inside the cage, the carbide cluster shows some degree of disorder: six existing La sites for the two La atoms and two disordered positions for the C_2_ unit (Cai et al., 2016). It appears that the defective *C*_2_-C_104_(816) cage appreciably hinders the free movement of the metal atoms when compared with the locations of La_2_C_2_@*D*_5_-C_100_(450) and La_2_C_2_@*C*_*s*_-C_102_(574) possessing ideal tubular cages (Cai et al., 2016). The pyracylene unit existing in the [10]cyclacene framework is responsible for this phenomenon (Cai et al., 2016). In addition, the predominant La ions in La_2_C_2_@*C*_2_-C_104_(816) are detached, and the line across them is slightly misaligned along the long axis of the carbon cage (Cai et al., 2016). As shown in Figure 12C′, the two major La ions in La_2_C_2_@*C*_2_-C_104_(816) depart from the pyracylene region, with one situated around a [6,6]-bond and the other located over a [5,6]-bond on the opposite side. Similarly, the La_2_C_2_ unit shows a stretched and nearly planar geometry, and the disordered C_2_ unit is no longer perpendicular to the line across the two major La ions (Cai et al., 2016). The La–C–C–La dihedral angle (157.5°) is much larger than that in La_2_C_2_@*D*_5_-C_100_(450) (141.3°), whereas and is less than that in La_2_C_2_@C_s_-C_102_(574) (173.6°) (Cai et al., 2016). The abnormally small value of the La–C–C–La dihedral angle in La_2_C_2_@*C*_2_-C_104_(816) may be attributed to the presence of the pyracylene “defect” destroying the ideal tubular structure (Cai et al., 2016).

So far, Y_2_C_2_@*C*_1_-C_108_(1660) has been the largest metallofullerene, with the linear configuration of the encapsulated carbide cluster characterized by crystallography, as shown in Figure 12D (Pan et al., 2018). The Vis-NIR absorption spectrum of Y_2_C_110_ showing absorption bands at 533, 654, 852, and 1,037 nm, with an onset at around 1,400 nm (Figure 10D), indicates that it has a small optical gap (0.89 eV) (Pan et al., 2018). Fortunately, the molecular structure of Y_2_C_110_ has been definitely confirmed by the crystallographic study of co-crystals of Y_2_C_2_@*C*_1_-C_108_(1660)·2Ni(OEP) (Pan et al., 2018). There are some degrees of disorder with respect to the metal atoms, showing 12 positions in all, with occupancies ranging from 0.080 to 0.204 (Pan et al., 2018). This endohedral fullerene is obliquely surrounded by two Ni(OEP) molecules, and the Ni–cage distances are 2.895 and 3.054 Å, which equal those for La_2_@*D*_5_-C_100_(450) and Sm_2_@*D*_3*d*_-C_104_(822) (Mercado et al., 2009; Beavers et al., 2011). Y_2_C_110_ is unambiguously assigned to a carbide cluster EMF, Y_2_C_2_@*C*_1_-C_108_(1660), utilizing an asymmetric chiral cage, which is one of the 1799 IPR isomers of C_108_ (Pan et al., 2018). Surprisingly, this cage has a relatively round shape as a result of the absence of a band of contiguous hexagons and more evenly distributed pentagons, which differs from the reported tubular giant EMF (Mercado et al., 2009; Beavers et al., 2011; Cai et al., 2016). Hence, Y_2_C_2_@*C*_1_-C_108_(1660) has a relatively short length (10.04 Å) compared with the other smaller giant cages (Pan et al., 2018). In particular, as shown in Figure 12D′, the Y_2_C_2_ cluster shows a linear configuration along the long axis of the carbon cage due to its ample inner space. This is the first experimental evidence of a linear M_2_C_2_ cluster that is coincident with the theoretical predictions of Dorn et al. (Zhang et al., 2012). The Y–C–C–Y dihedral angle is 173.1°, which indicates a linear configuration, while the C–C bond (1.08 Å) is shorter than a typical C≡C triple bond. The Y–Y distance of 5.85 Å is nearly equal to that of the free Y_2_C_2_ cluster (5.83 Å) suggested by theoretical calculations, but is obviously longer than the theoretical values for Y_2_C_2_ in Y_2_C_2_@*C*_3*v*_-C_82_(8) (3.74 Å), Y_2_C_2_@*D*_3_-C_92_(85) (4.92 Å), and Y_2_C_2_@*D*_5_-C_100_(450) (5.51 Å) (Zhang et al., 2012). This confirms experimentally that the compression of the encapsulated cluster induced by the fullerene cage can be ignored in Y_2_C_2_@*C*_1_-C_108_(1660) (Pan et al., 2018).

### Top-Down Formation Mechanisms

A top-down formation mechanism for endohedral fullerenes was put forward by Lu et al., who carefully analyzed the cage connectivity of reported giant fullerenes (Cai et al., 2016). Starting with the defective tubular cage *C*_2_-C_104_(816) (obtained as La_2_C_2_@*C*_2_-C_104_) (816), the other three ideal tubular cages, including *D*_5_-C_100_(450) (obtained as La_2_@*D*_5_-C_100_(450) and La_2_C_2_@*D*_5_-C_100_) (450), *C*_*s*_-C_102_(574) [obtained as La_2_C_2_@*C*_*s*_-C_102_(574)], and *D*_3*d*_-C_104_(822) [obtained as Sm_2_@*D*_3*d*_-C_104_(822)], can be achieved by elimination of the pyracylene motif or by an SW transformation (Cai et al., 2016).

**Figure 14:**
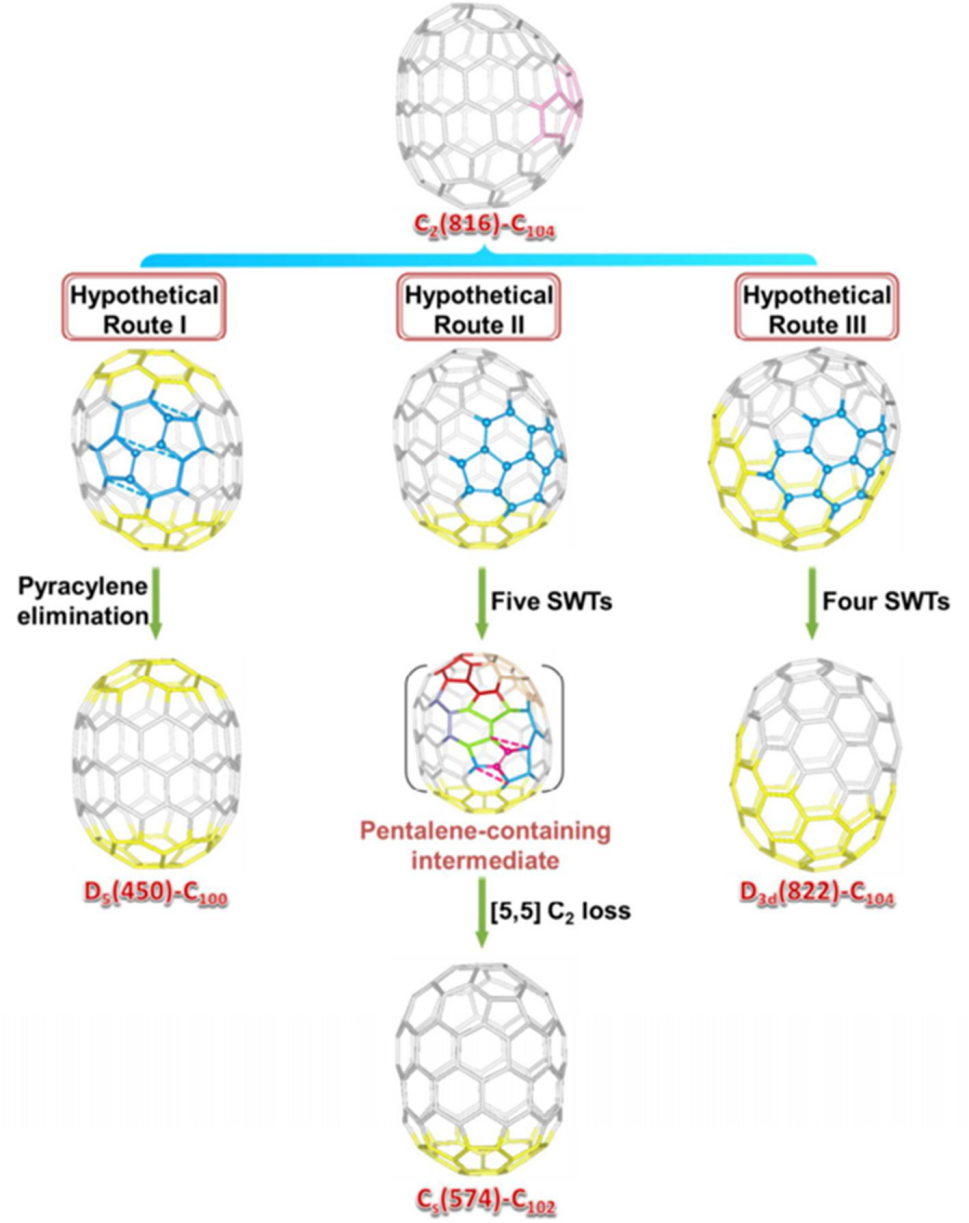
Rearrangement pathways from the defective *C*_2_-C_104_(816) cage to the other three ideal tubular fullerene cages, *D*_5_-C_100_(450), *C*_*s*_-C_102_(574), and *D*_3*d*_-C_104_(822). The starting point carbon atoms in the three rearrangement processes are highlighted in blue, whereas the unchanged caps are highlighted in yellow.

As shown in Figure 14, the rearrangement pathways where the partial regions of *C*_2_-C_104_(816) are found, and which are similar to the area of the target cages, are marked in yellow. Clearly, the two poles of *C*_2_-C_104_(816) are equal to those of *D*_5_-C_100_(450), whereas, the difference is from the pyracylene unit intercalated in the two [10]cyclacene layers. Hence, *D*_5_-C_100_(450) can be obtained from *C*_2_-C_104_(816) by a direct C_4_ loss from the pyracylene unit (Hypothetical Route I) (Cai et al., 2016).

The conversion from *C*_2_-C_104_(816) to *C*_*s*_-C_102_(574) is slightly complicated and follows Hypothetical Route II (Figure 14). An intermediate having one heptagon and a pair of fused pentagons is first formed by five SW transformation steps, starting with the elimination of the original pyracylene unit. Subsequently, a C_2_ loss from an indene unit sharing the pentalene pentagon leads to the formation of the *C*_*s*_-C_102_(574) (Cai et al., 2016).

The tubular *D*_3*d*_-C_104_(822) cage is obtained from *C*_2_-C_104_(816) via four SW transformation steps (Hypothetical Route III; Figure 14). The first step is an SWR2 rotation on a [6,6]-bond, which connects a hexagon and a pentagon. Such a bond rotation also occurs in the formation of a classical C_96_(*NC*3) cage from three heptagons (Cai et al., 2016).

Evidently, the defective tubular cage *C*_2_-C_104_(816) could be recognized as a starting point for other ideal tubular cages, as evidence for the “top-down” formation mechanism of fullerenes, whereas the “bottom-up” mechanism is an alternative (Cai et al., 2016). A similar case for the asymmetric *C*_1_-C_84_(51383) cage was put forward by Dorn et al. (Zhang et al., 2013). Consequently, the starting structure of the top-down formation mechanism is not merely restricted to non-IPR cages, but the defect fullerene, *C*_2_-C_104_(816), can also act as a “missing link.” (Cai et al., 2016).

## Conclusion and Prospects

### Key Factors for Determining Isomer Structures

From the above results, it is remarkable that the giant empty fullerenes always possess different cage isomers compared with the giant endohedral metallofullerenes. For example, the reported isomers of empty C_100_ cages are *C*_2_-C_100_(18) (Yang et al., 2014a), *D*_5*d*_-C_100_(1) (Fritz et al., 2014), *C*_1_-C_100_(425) (Wang et al., 2016a), and *C*_2*v*_-C_100_(417), (Wang et al., 2016a) whereas La_2_@C_100_ (Yang et al., 2008) and La_2_C_2_@C_100_ (Cai et al., 2015) utilize the *D*_5*h*_*-*(450) isomer cage. Current consensus is that charge transfer plays a crucial role in determining the isomeric structures of fullerenes. Briefly, when metal ions or clusters are encapsulated in a fullerene cage, charge transfer occurring between the embedded species and the carbon cage results in the carbon cage being negatively charged. As a consequence, the electronegative cage has a distinctly different stability compared with the neutral carbon cage, so that inconsistent isomers of empty and endohedral fullerenes are always produced. For the above-mentioned giant fullerene C_100_, theoretical calculations indicate that the *D*_5_-C_100_(450) cage is the most promising candidate for encapsulating a unit such as Sc_3_N or La_2_ with six electrons transferred, which has been confirmed by the findings for La_2_@*D*_5_-C_100_(450). On the other hand, La_2_@*D*_5_-C_100_(450) and La_2_C_2_@ *D*_5_-C_100_(450) have the same isomer cages, whereas their electronic configurations are probably different as a result of their significantly inconsistent absorption spectra. Four-electron transfer has generally been suggested for the bimetallic carbide, M_2_C_2_@C_2*n*_, as for the reported Y_2_C_2_@C_2*n*_ (Zhang et al., 2012), Sc_2_C_2_@C_2*n*_ (Zhang et al., 2012), Gd_2_C_2_@C_2*n*_ (Yang et al., 2008), and Tb_2_C_2_@C_2*n*_ (Liu et al., 2014). However, theoretical calculations for the four electronic configurations of the giant endohedral metallofullerenes suggest them to be vacant, while we can also speculate that other isomers of M_2_C_2_@C_100_ should exist.

In contrast, the electronic configuration of the reported Sm_2_@*D*_3*d*_-C_104_(822) is (Sm^2+^)_2_@[*D*_3*d*_-C_104_^4-^(822)], as suggested by the computational data (Mercado et al., 2009). La_2_C_2_@*C*_2_-C_104_(816) should adopt the same electronic configuration as [La_2_C_2_]^4+^@[*C*_2_-C_104_(816)]^4−^, provided that the convention is obeyed that four-electron transfer occurs for the bimetallic carbide fullerenes. According to this hypothesis, Sm_2_@*D*_3*d*_-C_104_(822) and La_2_C_2_@*C*_2_-C_104_(816) have the same electronic configurations, whereas they possess different carbon cages. A conclusion that the encapsulated cluster also influences the isomeric structure of the endohedral metallofullerenes could therefore be drawn. Briefly, two Sm ions having a greater separation distance preferentially occupy the longer cage isomer, *D*_3*d*_-C_104_(822), whereas the La_2_C_2_ cluster utilizes the relatively shorter cage isomer, *C*_2_-C_104_(816), though the La_2_C_2_ cluster is apparently more crowded. ^[19, 28]^However, further evidence needs to be provided to confirm this hypothesis.

Additionally, for endohedral fullerenes, the interactions between the cage and the cluster have been considered. In particular, for the carbide fullerene, the geometric configuration of the cluster has gradually evolved from the bent structure (Zhang et al., 2013) to the twisted structure (Cai et al., 2016), and finally to the nearly linear structure (Pan et al., 2018), which has been predicted by theoretical calculations and confirmed by the experimental data. Accordingly, the cage becomes larger in order to adapt to the change in clusters. In other words, the interactions between the cage and the cluster result in the ultimate geometric configurations of endohedral metallofullerenes.

### Prospects

Structural elucidations of giant fullerenes have been achieved thanks to exohedral chlorination, and giant endohedral fullerenes have also been investigated. In particular, using chlorination, not only has the connectivity of the carbon cage been studied but also novel fullerenes, including non-classical and non-IPR fullerenes have been studied. However, other giant fullerenes such as the empty giant fullerenes beyond C_108_, La_3_N@C_2*n*_(2*n*≥100) (Chaur et al., 2008), and especially M_3_@C_2*n*_(2*n*≥100) (Sarina et al., 2015) have not been experimentally identified by single-crystal X-ray diffraction. In particular, as the carbon cage becomes larger, the possibility of the inclusion of an M_3_ cluster within the giant cage increases, and M_3_C_2_@C_2*n*−2_ should also be considered as potential candidates.

Nevertheless, the properties of the giant empty fullerenes and endohedral metallofullerenes have scarcely been studied, even though a handful of giant fullerenes have been synthesized and isolated. Theoretical calculations indicate that empty giant fullerenes such as C_106_ possess outstanding optical non-linearity (Wang et al., 2015). Furthermore, the giant endohedral metallofullerenes are expected to be used in single-molecule devices. Therefore, further efforts should be made to promote the many different potential applications of giant fullerenes.

## Author Contributions

SW, SY, and ST discussed the outline. QC and GZ collected the literatures and plotted the figures. SW wrote the first draft. XW and FL edited the manuscript. SW, SY, and ST reviewed the manuscript. All authors contributed to the article and approved the submitted version.

## Conflict of Interest

The authors declare that the research was conducted in the absence of any commercial or financial relationships that could be construed as a potential conflict of interest.
